# A new group of active impedance source inverters with lower components and voltage stress across active switches

**DOI:** 10.1038/s41598-026-40820-z

**Published:** 2026-02-27

**Authors:** Vida Ranjbarizad, Ebrahim Babaei, Soheil Salahshour

**Affiliations:** 1 Faculty of Electrical and Computer Engineering, University of Tabriz, Tabriz, Iran; 2 Faculty of Engineering and Natural Sciences, Istanbul Okan University, Istanbul, Turkey; 3 Energy Systems Research Center, Khazar University, Mahasti Str. 41, Baku, AZ1096 Azerbaijan

**Keywords:** Energy science and technology, Engineering

## Abstract

This paper proposes a new group of active impedance source inverters along with an appropriate pulse-width modulation (PWM) control method. These inverters have reduced voltage stress across capacitors, diodes, and active switches in comparison to conventional topologies. The paper provides a detailed explanation of the control method used to generate gate pulses for the switches, as well as the analysis of all operating modes. The calculation of the boost factor and the design of capacitors and inductors are presented. A comparative analysis is conducted from various perspectives, such as the boost factor, the total stress voltages on active switches, diodes, capacitors, the volume of passive components, and the efficiency of proposed and conventional topologies. The results show better performance for the proposed topologies compared to conventional topologies. Finally, the experimental results from one prototype validate the analytical findings.

## Introduction

Today, inverters play a critical role in industrial applications such as motor drives [1], power supplies [2], distributed power systems [3], electric vehicles [4], etc. Therefore, they are widely employed in industrial systems. Initially, the conventional voltage source inverters (VSIs) were used. However, they had numerous drawbacks, such as buck operation, the requirement for dead time to ensure safe commutation of power switches, and electromagnetic interference (EMI). An additional DC–DC boost converter must be inserted to eliminate the buck-type operation of VSIs. Furthermore, to address the dead time and EMI issues, Z source inverter (ZSI) has been introduced [5]. The conventional ZSI has some problems, such as discontinuous input current, high capacitor voltage, no common ground with the source, and large inrush current during startup. To overcome these limitations, quasi-ZSI (qZSI) [6], improved ZSI [7], and inductor-ZSI (L-ZSI) [8] have been presented. However, their boost factors are the same as those of conventional ZSI. To increase the boost factor, switched inductor cells (SLs) are replaced with the inductors in the conventional ZSI and qZSI ([9, 10]). Switched capacitor cells (SCs) are another way to increase the boost factor of ZSIs [11]. Some topologies, including SLs and SCs, are used to significantly increase the boost factor [12, 13]. However, SL- and SC-based topologies increase the number of passive components, which eventually increases the inverter’s weight, volume, and size. Coupled inductor and transformer-based topologies ([14–16]) are presented to increase the boost factor. Further, the leakage inductance of the coupled inductors and transformer increases power losses and decreases efficiency. Cascading different topologies can also achieve an improved boost factor [17]. However, this method requires a large number of elements.

Over the past decade, numerous modifications of the ZSI have been introduced to overcome its inherent limitations, such as the high number of passive components, large voltage stress across elements, and discontinuous input current. One early solution is the switched boost inverter (SBI) introduced in [18], which adds an active switch to the impedance network of the conventional ZSI. This change reduces the number of passive components, thus lowering the inverter’s size and weight. However, SBI suffers from a limited boost factor and a discontinuous input current, which constrain its performance in practical applications. To improve the boost capability, reduce the number of passive components, lower voltage stress across elements, and maintain continuous input current, several enhanced topologies have been proposed in [19–36]. For example, in [19], the active switched capacitor/switched inductor quasi-Z-source inverter (ASC/SL-qZSI) integrates SCs and SLs into the SBI structure, resulting in a higher boost factor, although the topology still suffers from the same problems as topologies with SLs and SCs. Similarly, [20] combines the SBI and qZSI to create a topology that provides a high voltage gain with fewer passive components. However, this design lacks a common ground between the source and inverter bridge, involves more active switches, and introduces high voltage stress across capacitors. In [21], a new topology based on switched Z-impedance is presented to achieve a high boost factor. While it reduces complexity, it still suffers from discontinuous input current, high capacitor voltage stress, lack of common ground, and inrush current at startup. To address some of these drawbacks, the topology in [22] improves the boost factor and input current continuity. However, it uses four inductors and four capacitors, which increases the size, weight, and cost of the inverter. An improved version, the enhanced-boost-qZSI with an active switched Z-network, is proposed in [23]. This design achieves a similar high boost factor to [22] but reduces two LC pairs by introducing an active switch. It also offers continuous input current and common ground. Nevertheless, it still suffers from high voltage stress across passive and active components. To achieve even higher voltage gains, a multi-cell ASC/SL-qZSI is proposed in [24], which extends the basic structure from [19] by adding *n* capacitor-based and *m* inductor-based cells. Although the boost factor improves, the inverter faces significant drawbacks due to the high voltage stress and increased number of elements. A different approach is taken in [25], where a three-winding coupled-inductor network is introduced. The inverter achieves flexible voltage gain through varying turn ratios. However, the use of coupled inductors introduces leakage inductance, which reduces efficiency and increases the total blocked voltage across diodes. Further efforts in [26] combine SBI with both SC and SL cells, resulting in two new topologies with extremely high boost factors. Yet, these designs use a high number of components, which compromise size and efficiency. In [27], a high-gain active-SB-ZSI is introduced, featuring a novel PWM strategy to reduce voltage stress on semiconductor devices. The proposed topology employs an advanced impedance network to achieve a significant boost in voltage gain while maintaining improved reliability and reduced stress across active components. Similarly, in [28], an enhanced high-boost active-switched-qZSI is presented that achieves high voltage gain with a shorter range of shoot-through duty ratio, making it particularly suitable for solar energy conversion systems. Although both topologies improve voltage boosting and help reduce voltage stress, they still require many components, which adds to the system’s size, cost, and complexity.

With changes in the number and position of components in qZSI, particularly through the incorporation of SLs and SCs, the topologies in [29] are introduced. These configurations demonstrate a significant improvement in voltage boost capability, primarily due to the strategic arrangement of SL and SC cells. However, they require a higher number of passive components, which increases the system’s size, complexity, and cost. Similarly, in [30], an ultra-high-gain qZSI that employs an active switched network to achieve an exceptionally high voltage gain is proposed. This structure effectively extends the shoot-through interval and enhances energy transfer efficiency. Nevertheless, it suffers from very high voltage stress across both active and passive components, as well as increased circuit complexity due to the large number of switching elements. In [31], a coupled-inductor-assisted high voltage gain half-bridge ZSI is proposed, which utilizes T-shaped coupled inductors to achieve a significantly increased boost factor without using transformers or requiring extreme shoot-through durations. This approach improves voltage gain flexibility by adjusting the turn ratios of the coupled inductors. However, it introduces inherent challenges, such as leakage inductance, increased diode voltage stress, and reduced overall efficiency—issues that limit its suitability for compact and high-efficiency power conversion applications. Meanwhile, in [32], a single-phase high step-up active-switched quasi-Z-source neutral-point-clamped (NPC) inverter is introduced, featuring a common ground structure, enabling safer and more efficient integration with low-voltage sources. This topology combines active switched networks and an NPC structure to achieve high voltage gain and improved modularity, but it comes at the cost of a large number of passive and active elements, resulting in increased volume, system complexity, and implementation cost. In recent years, published papers have mostly focused on voltage boosting. For example, in papers [33] and [34], a voltage-lifting SL cell has been used. This cell increases the voltage gain, but it still retains the issues associated with circuits that employ this cell.

As observed, most of the proposed inverters with high voltage gain still face issues such as increased voltage stress across components and a large number of elements. Therefore, designs that can offer sufficient voltage gain while reducing voltage stress, lowering the number of components, improving efficiency, and simplifying the circuit structure remain important. Accordingly, several topologies aimed at meeting these objectives have been presented in [35–38]. In [35], the embedded active ZSI is introduced, which integrates the impedance source network more efficiently into the inverter structure. This topology offers a simplified configuration and reduces the number of passive elements compared to traditional ZSI, while maintaining acceptable voltage gain performance. However, its boost capability remains moderate, and certain limitations, such as discontinuous input current and voltage stress across specific components, still exist. Expanding on this research, the authors of [36] introduced a new type of active ZSI that is specially designed to reduce voltage stress on semiconductor devices. This design greatly enhances reliability and efficiency by optimizing the impedance network and smartly using active components within the circuit. Despite these improvements, the designs in both [35] and [36] still face constraints in terms of scalability of voltage gain and require a high duty cycle for applications demanding very high boost factors. Also, [37] introduce a new topology that improve boost factor and reduce the voltage stress across components but investigated only a single impedance-source inverter topology and was limited to extracting basic operational parameters through simulation. It did not provide a comprehensive structural comparison, detailed loss and dynamic analyses, wide-range efficiency evaluation, or any experimental validation. Another article presented in [38] shows that by applying a new PWM control method, the voltage stress across elements can be reduced. This modulation strategy focuses on reducing voltage stress and inductor current ripple in a qZSI. However, the control method still faces challenges in achieving stable continuous inductor current and balancing voltage stress across the switches. Also, the control complexity is high and sensitive to load changes.

In this paper, the limitations reported in earlier studies are addressed, and the main contributions of this work are listed as follows:

- Five converter topologies are proposed, each designed to reduce the component count, decrease device voltage stress, and improve efficiency, thereby addressing the limitations reported in previous works [18]–[35].
- A complete control strategy for the proposed structures is developed and explained in detail.
- All operating modes are analyzed comprehensively, and the corresponding equations are compiled in a dedicated summary table.
- Design procedures for active and passive components are derived to support practical implementation.
- A comprehensive comparative study is performed between the proposed topologies and several conventional topologies from multiple performance perspectives such as voltage gain, voltage stress across all elements, power density, efficiency.
- Small-signal models are developed to evaluate the dynamic behavior and stability of the converters.
- Transient-state waveforms are presented to demonstrate the time-domain response under various operating conditions.
- A hardware prototype is built, and the experimental results validate the analytical and simulation outcomes.

## Proposed a new group of active impedance source inverters

**Fig. 1:**
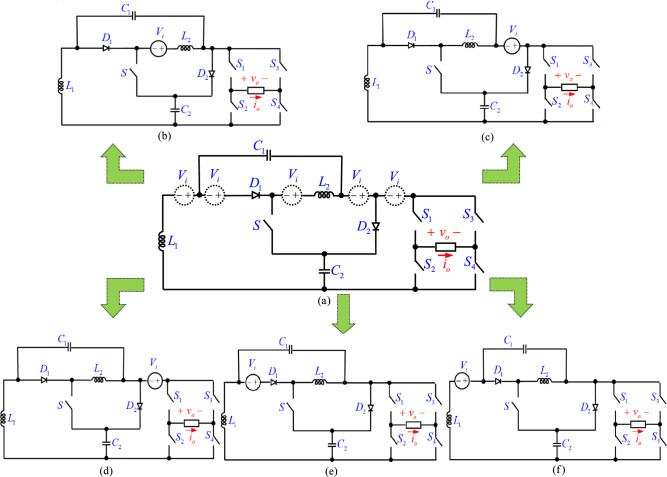
(**a**) Schematic of a new class of impedance source inverters, (**b**) PT1, (**c**) PT2; (**d**) PT3; (**e**) PT4; (**f**) PT5.

Figure 1 shows the power circuits of the proposed impedance source inverters. As this figure shows, similar to other ZSIs, these inverters consist of two sides. The first side is an H-bridge unit and the second side is an impedance unit. The H-bridge unit consists of switches *S*_*1*_, *S*_*2*_, *S*_*3*_, and *S*_*4*_. Here, the single-phase inverter is used at the output side. For three-phase applications, it is sufficient to replace the single-phase H-bridge with a three-phase inverter. The impedance unit contains an active impedance network which performs as a DC/DC converter. The impedance network consists of two cells. The first cell uses *L*_*1*_, *C*_*1*_, and *D*_*1,*_ and the second cell uses *L*_*2*_, *C*_*2*_, and *D*_*2*_. At the impedance unit, apart from these cells, a switch (*S*) is also used. By having a DC voltage source in different locations, five different topologies can be extracted as shown in Fig. 1. These topologies are named PT1, PT2, PT3, PT4, and PT5 (first, second, third, fourth, and fifth proposed topologies, respectively). In the following, only PT1 is analyzed in detail, and for the other topologies, only the results are presented.

The H-bridge unit has three operating modes. In the first operating mode, *S*_*1*_ and *S*_*4*_ are on, in the second operating mode, *S*_*2*_ and *S*_*3*_ are on, and finally in the third operating mode, either one leg or all switches are on. The first and second operating modes are known as non-shoot through state (non-ST), and the third operating mode is known as shoot through (ST) state. The switch *S* is turned on during the ST time interval and is turned off during the non-ST time interval. During ST time interval, the diodes *D*_1_ and *D*_2_ are turned off due to having negative voltages. In non-ST state, the opposite situation occurs. Also, the duty cycle (*D*_*ST*_) represents the time interval during which the switches operate in the ST mode within a single switching period. It defines the duration of conduction for the switches in each cycle and directly influences the converter’s performance.

### Presented control method

**Fig. 2:**
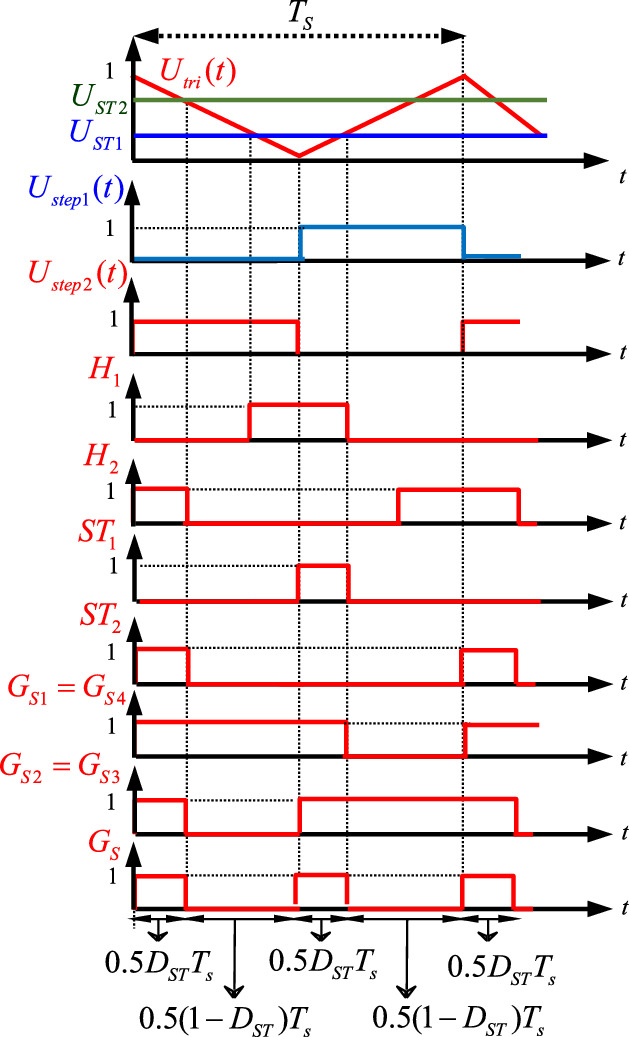
Generation of trigger pulses by using the presented control method.

To generate the required trigger pulses, the presented control method in Fig. 2 can be used. To control the proposed topology, three gate signals for switches (*S*_*1*_, *S*_4_), (*S*_2_, *S*_3_), and *S* are needed. To generate these signals, two constant reference signals (*U*_*ST*1_ and *U*_*ST*2_) and a carrier signal (*U*_*tri*_) are used. The carrier signal has triangular shape with frequency equal to switching frequency. Moreover, two square waveforms *U*_*step1*_ and *U*_*step2*_ as shown in Fig. 2 are considered. By comparing reference and carrier signals, *H*_1_ and *H*_2_ are generated. If *U*_*ST1*_ is more than triangular signal, in this case *H*_1_ will be equal to 1 and otherwise will be equal to zero. By the same method, If *U*_*ST*2_ is lower than triangular signal, in this case *H*_2_ will be equal to 1 and otherwise will be equal to zero. Whenever both *U*_*step*1_ and *H*_1_ are simultaneously equal to 1, the signal *ST*_1_ is considered to be 1. Similarly, whenever both *U*_*step*2_ and *H*_2_ are simultaneously equal to 1, the signal *ST*_2_ will be equal to 1. Then, either *U*_*step*2_ or *ST*_1_ are equal to 1, the gate signals for switches *G*_*S*1_ and *G*_*S*4_ are generated (*G*_*S*1_ = *G*_*S*4_). By the same method, either *U*_*step*1_ or *ST*_2_ are equal to 1, the gate signals for switches *G*_*S*2_ and *G*_*S*3_ are produced (*G*_*S*2_ = *G*_*S*3_). At the end, whenever both *G*_*S*1_ and *G*_*S*2_ are simultaneously equal to 1, the required signal for switch *S* is obtained (*G*_*S*_).

The proposed control method can be applied to a three-phase inverter by repeating the same logic for each of the three legs, but with a timing shift so that the waveforms for phase *B* are delayed by 120 degrees and those for phase *C* are delayed by 240 degrees compared to phase *A*. This phase shift ensures that the shoot-through periods for each leg happen at the right time in relation to their own modulating signals.

In each phase, the reference signal is compared with the carrier, and together with *U*_*step1A*_ and *U*_*step2A*_ (shifted by 120° and 240° for phases *B* and *C*, respectively.), the same AND/OR logic is applied to generate the gating pulses and the ST states.

## Analysis of first proposed topology

**Fig. 3:**
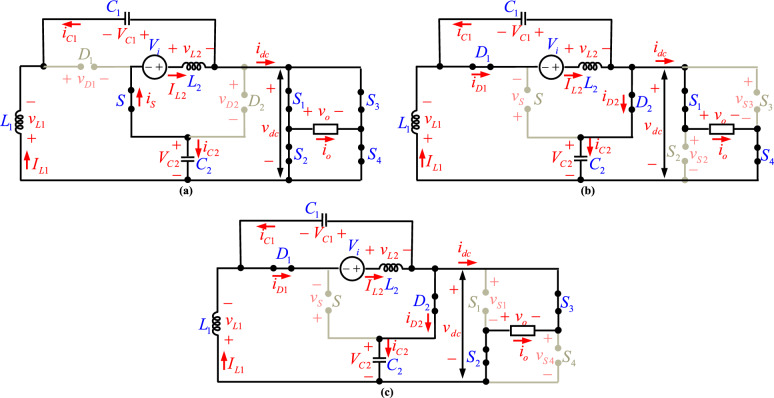
The equivalent circuits of the proposed topology at different operating modes; (**a**) first and third, (**b**) second, (**c**) fourth.

In this section, PT1 in different operating modes is analyzed in detail, and the basic equations in all operating modes are extracted. The equivalent circuits of this topology in different operating modes are shown in Fig. 3.

### First operating mode ($0 < t < 0.5\,D_{ST} T_{s} \,$ or [t_1_])

In the first operating mode as shown in Fig. 3a, all switches (*S*_1_,$\cdots$, *S*_4_, and *S*) are on and all diodes (*D*_*1*_ and *D*_*2*_) are off. This operating mode is named by ST state. By using KVL, the voltages across diodes in the ST state can be obtained:

1 $$ v_{D1} = - v_{L1} - v_{C2} $$

2 $$ v_{D2} = - v_{C2} $$

By Applying KCL, the current through capacitors can be obtained as:

3 $$ i_{C1} = - I_{L1} $$

4 $$ i_{C2} = - I_{L2} $$

The voltages across inductors are calculated by:

5 $$ v_{L2} = V_{i} + V_{C2} $$

6 $$ v_{L1} = V_{C1} $$

Equations (5) and (6) indicate positive inductor voltages, meaning the inductors charge during this interval. The current of the DC-link is given by:

7 $$ I_{dc} = I_{L2} - i_{C1} $$

In this operating mode, the output voltage is zero because the switches of the H-bridge cell are on.

###  Second operating mode ($0.5D_{ST} T_{s} < t < 0.5T_{s}$ or [t_2_])

The equivalent of this operating mode is shown in Fig. 3b. As this figure shows, the switches *S*_1_ and *S*_4_ are turned on to produce a positive output voltage. Also, the switches *S*_2_, *S*_3*,*_ and *S* are turned off, and diodes *D*_1_ and *D*_2_ are in forward bias. In this operating mode, the currents through capacitors are increased and the voltages across inductors are decreased. Here, the following equations can be written:

8 $$ i_{C1} = I_{L2} - I_{L1} $$

9 $$ i_{C2} = I_{L1} - i_{O} $$

10 $$ v_{L1} = V_{C1} - V_{C2} $$

11 $$ v_{L2} = V_{i} - V_{C1} $$

12 $$ V_{dc,\max } = V_{o,\max } = V_{C2} $$

### Third operating mode ($0.5T_{s} < t < 0.5(1 + D_{ST} )T_{s}$ or [t_3_])

The equivalent circuits of the first and third operating modes are the same. So, all the first operating mode equations are valid for the third operating mode.

###  Fourth operating mode ($0.5(1 + D_{ST} )T_{s} < t < T_{s}$ or [t_4_])

According to Fig. 3c, the switches *S*_*2*_ and *S*_*3*_ are turned on to produce a negative output voltage. Also, the switches *S*_1_, *S*_4_, and *S* are turned off, and diodes *D*_1_ and *D*_2_ are in forward bias. Due to the similarity of the equivalent circuits of impedance network in both second and forth operating modes, the Eqs. (8)-(11) are valid for this operating mode. For output voltage, we have:

13 $$ v_{o} = V_{o,\min } = - V_{dc,\max } $$

**Fig. 4:**
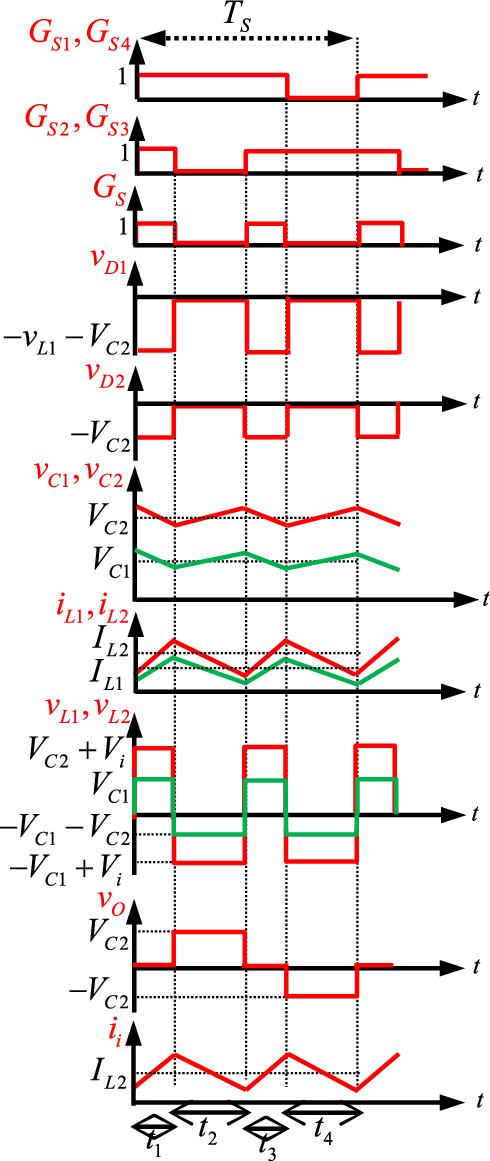
Steady-state key waveforms of PT1.

According to the analysis of the proposed topology in different operating modes, the key waveforms at steady-state during one period are shown in Fig. 4.

### Calculation of boost factor

The boost factor is defined as a ratio of maximum voltage on DC-link (*v*_*dc*_) to input voltage (*V*_*i*_). A principle named the volts-second law is used to obtain the boost factor. According to this law, the following equation can be written:

14 $$ \int\limits_{0}^{{T_{s} }} {v_{L} dt = 0} $$

By applying the above equation for inductors *L*_1_ and *L*_2_, and considering (5), (6), (10), and (11), the following equations are obtained:

15 $$ V_{C1} D_{ST} + (V_{C1} - V_{C2} )(1 - D_{ST} ) = 0 $$

16 $$ (V_{C2} + V_{i} )D_{ST} + (V_{i} - V_{C1} )(1 - D_{ST} ) = 0 $$

From the above equations, the average voltages across capacitors can be calculated by:

17 $$ V_{C1} = (1 - D_{ST} )V_{C2} $$

18 $$ V_{C2} = \frac{{V_{i} }}{{D_{ST}^{2} - 3D_{ST} + 1}} $$

By using (12) and (18), the values of maximum DC-link voltage and boost factor can be obtained:

19 $$ \begin{gathered} V_{dc.\max } = \frac{1}{{D_{ST}^{2} - 3D_{ST} + 1}}V_{i} = BV_{i} \hfill \\ B = \frac{1}{{D_{ST}^{2} - 3D_{ST} + 1}} \hfill \\ \end{gathered} $$

where, *B* is a boost factor.

Considering (12) and (13), we can write:

20 $$ V_{o,\max } = - V_{o,\min } = \frac{{V_{i} }}{{D_{ST}^{2} - 3D_{ST} + 1}} $$

### Design considerations

This section is intended for the designs of the values of passive (inductors and capacitors) and active (switches and diodes) elements. The ripple value of the inductors’ current is necessary to get the values of the inductors. The formula $v_{L} = Ldi_{L} /dt$ is used to obtain this ripple value. At first, we need to calculate the voltage of the inductors in one operating mode. For this reason, the first operating mode is used. Then, according to the above-mentioned formula and by using (5) and (6), we have:

21 $$ V_{C2} + V_{i} = 2L_{1} \frac{{{ Delta }i_{L1} }}{{D_{ST} T_{S} }} $$

22 $$ V_{C1} = 2L_{2} \frac{{{ Delta }i_{L2} }}{{D_{ST} T_{S} }} $$

Considering (17) and (18), the current ripple values of *L*_1_ and *L*_2_ are calculated as:

23 $$ { Delta }i_{L1} = \left| {\frac{{(D_{ST}^{2} - 3D_{ST} + 2)D_{ST} }}{{2L_{1} f_{S} (D_{ST}^{2} - 3D_{ST} + 1)}}V_{i} } \right| $$

24 $$ { Delta }i_{L2} = \left| {\frac{{(1 - D_{ST} )D_{ST} }}{{2L_{2} f_{S} (D_{ST}^{2} - 3D_{ST} + 1)}}V_{i} } \right| $$

Equations (23) and (24) show that higher switching frequency and inductance reduce inductor current ripple.

In the next step, the voltage ripple of capacitors should be calculated. For this reason, the formula $i_{C} = Cdv_{C} /dt$ can be used. As in the previous case, in the first operating mode by using this formula, (3), and (4), the ripple values of capacitors’ voltages are given by:

25 $$ { Delta }v_{C1} = \frac{{D_{ST} T_{S} I_{L1} }}{{2C_{1} }} $$

26 $$ { Delta }v_{C2} = \frac{{D_{ST} T_{S} I_{L2} }}{{2C_{2} }} $$

The above equations show the average inductors’ currents play a fundamental role in determining capacitors voltages’ ripple. So, in the following, the average values of inductors’ currents are calculated. A principle called ampere-second law is applied to calculate these values. According to this law, the average current value of a capacitor in one period is equal to zero. In other words:

27 $$ \int\limits_{0}^{{T_{s} }} {i_{C1} } = \int\limits_{0}^{{T_{s} }} {i_{C2} } = 0 $$

By using (3), (4), (8), and (9), the above equation can be rewritten as follows:

28 $$ - I_{L1} D_{ST} T_{S} + (1 - D_{ST} )(I_{L2} - I_{L1} )T_{S} = 0 $$

29 $$ - I_{L2} D_{ST} T_{S} + (1 - D_{ST} )(I_{L1} - i_{O} )T_{S} = 0 $$

By substituting (20) in the above equation, the values of the inductors’ currents are calculated as follows:

30 $$ I_{L1} = \frac{{(1 - D_{ST} )^{2} V_{i} }}{{R(D_{ST}^{2} - 3D_{ST} + 1)^{2} }} $$

31 $$ I_{L2} = \frac{{(1 - D_{ST} )V_{i} }}{{R(D_{ST}^{2} - 3D_{ST} + 1)^{2} }} $$

where, *R* is the ohmic value of output load.

By replacing the above equations in (25) and (26), the capacitors’ voltages ripples are obtained by:

32 $$ \Delta V_{C1} = \left| {\frac{{D_{ST} (1 - D_{ST} )^{2} V_{i} }}{{2RC_{1} f_{S} (D_{ST}^{2} - 3D_{ST} + 1)^{2} }}} \right| $$

33 $$ \Delta V_{C2} = \left| {\frac{{D_{ST} (1 - D_{ST} )V_{i} }}{{2RC_{2} f_{S} (D_{ST}^{2} - 3D_{ST} + 1)^{2} }}} \right| $$

The acceptable values of inductors’ currents ripples and capacitors’ voltages ripples in percentage are required to determine suitable values of capacitances and inductances. So, by having equations $\left| {\Delta i_{L} } \right| = x_{L} \% \,\,I_{L}$ and $\left| {\Delta v_{C} } \right| = x_{C} \% \,\,V_{C}$, and by replacing the acceptable values of inductors’ currents ripples and capacitors’ voltages ripples in percentage, the following equations are written:

34 $$ L_{1} = \frac{{D_{ST} (D_{ST} - 2)(D_{ST}^{2} - 3D_{ST} + 1)R}}{{2f_{S} (1 - D_{ST} )x_{L1} \% }} $$

35 $$ L_{2} = \frac{{D_{ST} (D_{ST}^{2} - 3D_{ST} + 1)R}}{{2f_{S} x_{L2} \% }} $$

36 $$ C_{1} = \frac{{D_{ST} (1 - D_{ST} )}}{{2Rf_{S} (D_{ST}^{2} - 3D_{ST} + 1)x_{C1} \% }} $$

37 $$ C_{2} = \frac{{D_{ST} (1 - D_{ST} )}}{{2Rf_{S} (D_{ST}^{2} - 3D_{ST} + 1)x_{C2} \% }} $$

At the following, all operating modes of the first proposed topology are analyzed, and the maximum voltage and current of semiconductor-based elements are calculated. Subsequently, in Table 1, the nominal values of those elements are shown. In order to simplify the table and avoid repeating certain equations, we use $A = D_{ST}^{2} - 3D_{ST} + 1$ as an abbreviation. According to this table, the maximum blocked voltages by diodes *D*_1_ and *D*_2_ are 1.76 and 1 times of the output voltage, respectively, at a duty cycle of 0.24. Additionally, the currents flowing through diodes *D*_1_ and *D*_2_ are 2.25 and 0.71 times of the output current, respectively. Similarly, at the same duty cycle, the maximum voltage across the active switch and the current flowing through it are found to be 0.76 times the output voltage and 2.25 times the output current, respectively. Also, the maximum voltage and current of the inverter-side switches are equal to the output voltage and output current, respectively.

**Table 1:** The maximum voltages and currents of semiconductor based elements for PT1.

Active components	Voltage	Current
$D_{1}$	$\frac{{2 - D_{ST} }}{A}V_{i}$	$\frac{{(1 - D_{ST} )V_{i} }}{{R(A)^{2} }}$
$D_{2}$	$\frac{1}{A}V_{i}$	$\frac{{D_{ST} V_{i} }}{{R(A)^{2} }}$
$S$	$\frac{{1 - D_{ST} }}{A}V_{i}$	$\frac{{(1 - D_{ST} )V_{i} }}{{R(A)^{2} }}$
$S_{1} = \cdots = S_{4}$	$\frac{1}{A}V_{i}$	$\frac{{V_{i} }}{(A)R}$

### Results for all proposed topologies

In this sub-section, all previous steps are repeated for the other proposed topologies as well, and key equations and design parameters for the proposed topologies are summarized in Table 2. In other words, in this table, the equations of the boost factor, the voltages across capacitors, the currents through inductors, the values of the passive elements (inductors and capacitors) and the active elements (diodes and switches) for all proposed topologies are shown.

**Table 2:** Results for proposed topologies.

	PT1	PT2	PT3	PT4	PT5
*B*	$\frac{1}{A}$	$\frac{1}{A}$	$\frac{{1 - D_{ST} }}{A}$	$\frac{{1 - D_{ST} }}{A}$	$\frac{{1 - D_{ST} }}{A}$
$V_{C1}$	$\frac{{1 - D_{ST} }}{A}V_{i}$	$\frac{{D_{ST} (2 - D_{ST} )}}{A}V_{i}$	$\frac{{D_{ST} }}{A}V_{i}$	$\frac{{(1 - D_{ST} )^{2} }}{A}V_{i}$	$\frac{{D_{ST} }}{A}V_{i}$
$V_{C2}$	$\frac{1}{A}V_{i}$	$\frac{1}{A}V_{i}$	$\frac{{D_{ST} (2 - D_{ST} )}}{A}V_{i}$	$\frac{{1 - D_{ST} }}{A}V_{i}$	$\frac{{1 - D_{ST} }}{A}V_{i}$
$I_{L1}$	$\frac{{(1 - D_{ST} )^{2} V_{i} }}{{R(A)^{2} }}$	$\frac{{(1 - D_{ST} )^{2} V_{i} }}{{R(A)^{2} }}$	$\frac{{(1 - D_{ST} )^{2} V_{i} }}{{R(A)^{2} }}$	$\frac{{(1 - D_{ST} )^{2} V_{i} }}{{R(A)^{2} }}$	$\frac{{(1 - D_{ST} )^{2} V_{i} }}{{R(A)^{2} }}$
$I_{L2}$	$\frac{{(1 - D_{ST} )V_{i} }}{{R(A)^{2} }}$	$\frac{{(1 - D_{ST} )V_{i} }}{{R(A)^{2} }}$	$\frac{{(1 - D_{ST} )V_{i} }}{{R(A)^{2} }}$	$\frac{{(1 - D_{ST} )V_{i} }}{{R(A)^{2} }}$	$\frac{{(1 - D_{ST} )V_{i} }}{{R(A)^{2} }}$
$C_{1}$	$\frac{{D_{ST} (1 - D_{ST} )}}{{2Rf_{S} (A)x_{C1} \% }}$	$\frac{{D_{ST}^{2} - 2D_{ST} + 1}}{{Rf_{S} (D_{ST}^{3} - 5D_{ST}^{2} + 7D_{ST} - 2)x_{C1} \% }}$	$\frac{{(1 - D_{ST} )^{2} }}{{2Rf_{S} (A)x_{C1} \% }}$	$\frac{{D_{ST} }}{{2Rf_{S} (A)x_{C1} \% }}$	$\frac{{(1 - D_{ST} )^{2} }}{{2Rf_{S} (A)x_{C1} \% }}$
$C_{2}$	$\frac{{D_{ST} (1 - D_{ST} )}}{{2Rf_{S} (A)x_{C2} \% }}$	$\frac{{D_{ST} (1 - D_{ST} )}}{{Rf_{S} (A)x_{C2} \% }}$	$\frac{{(1 - D_{ST} )}}{{2Rf_{S} (A)(D_{ST} - 2)x_{C2} \% }}$	$\frac{{D_{ST} }}{{2Rf_{S} (A)x_{C2} \% }}$	$\frac{{D_{ST} }}{{2Rf_{S} (A)x_{C2} \% }}$
$L_{1}$	$\frac{{D_{ST} (D_{ST} - 2)(A)R}}{{2f_{S} (1 - D_{ST} )x_{L1} \% }}$	$\frac{{D_{ST} R(A)}}{{2f_{S} x_{L2} \% (1 - D_{ST} )}}$	$\frac{{D_{ST} (A)R}}{{2f_{S} x_{L1} \% }}$	$\frac{{D_{ST} (A)R}}{{2f_{S} x_{L1} \% }}$	$\frac{{D_{ST} (A)R}}{{2f_{S} x_{L1} \% }}$
$L_{2}$	$\frac{{D_{ST} (A)R}}{{2f_{S} x_{L2} \% }}$	$\frac{{D_{ST} (A)(2 - DST)R}}{{2f_{S} x_{L2} \% }}$	$\frac{{D_{ST} (A)R}}{{2f_{S} x_{L2} \% }}$	$\frac{{D_{ST} (A)R}}{{2f_{S} x_{L2} \% }}$	$\frac{{D_{ST} (A)R}}{{2f_{S} x_{L2} \% }}$
$V_{D1}$	$\frac{{2 - D_{ST} }}{A}V_{i}$	$\frac{{2 - D_{ST} }}{A}V_{i}$	$\frac{1}{A}V_{i}$	$\frac{1}{A}V_{i}$	$\frac{1}{A}V_{i}$
$V_{D2}$	$\frac{1}{A}V_{i}$	$\frac{1}{A}V_{i}$	$\frac{{1 - D_{ST} }}{A}V_{i}$	$\frac{{1 - D_{ST} }}{A}V_{i}$	$\frac{{1 - D_{ST} }}{A}V_{i}$
$V_{S}$	$\frac{{1 - D_{ST} }}{A}V_{i}$	$\frac{{1 - D_{ST} }}{A}V_{i}$	$\frac{{D_{ST} }}{A}V_{i}$	$\frac{{D_{ST} }}{A}V_{i}$	$\frac{{D_{ST} }}{A}V_{i}$
$V_{S1} = \cdots = V_{S4}$	$\frac{1}{A}V_{i}$	$\frac{1}{A}V_{i}$	$\frac{{1 - D_{ST} }}{A}V_{i}$	$\frac{{1 - D_{ST} }}{A}V_{i}$	$\frac{{1 - D_{ST} }}{A}V_{i}$
$i_{D1}$	$\frac{{(1 - D_{ST} )V_{i} }}{{R(A)^{2} }}$	$\frac{{(1 - D_{ST} )V_{i} }}{{R(A)^{2} }}$	$\frac{{(1 - D_{ST} )V_{i} }}{{R(A)^{2} }}$	$\frac{{(1 - D_{ST} )V_{i} }}{{R(A)^{2} }}$	$\frac{{(1 - D_{ST} )V_{i} }}{{R(A)^{2} }}$
$i_{D2}$	$\frac{{D_{ST} V_{i} }}{{R(A)^{2} }}$	$\frac{{D_{ST} V_{i} }}{{R(A)^{2} }}$	$\frac{{(1 - D_{ST} )D_{ST} (2 - D_{ST} )V_{i} }}{{R(A)^{2} }}$	$\frac{{(1 - D_{ST} )D_{ST} (2 - D_{ST} )V_{i} }}{{R(A)^{2} }}$	$\frac{{(1 - D_{ST} )D_{ST} (2 - D_{ST} )V_{i} }}{{R(A)^{2} }}$
$i_{S}$	$\frac{{(1 - D_{ST} )V_{i} }}{{R(A)^{2} }}$	$\frac{{(1 - D_{ST} )V_{i} }}{{R(A)^{2} }}$	$\frac{{(1 - D_{ST} )V_{i} }}{{R(A)^{2} }}$	$\frac{{(1 - D_{ST} )V_{i} }}{{R(A)^{2} }}$	$\frac{{(1 - D_{ST} )V_{i} }}{{R(A)^{2} }}$
$i_{S1} = \cdots = i_{S4}$	$\frac{{V_{i} }}{(A)R}$	$\frac{{V_{i} }}{(A)R}$	$\frac{{1 - D_{ST} }}{(A)R}V_{i}$	$\frac{{1 - D_{ST} }}{(A)R}V_{i}$	$\frac{{1 - D_{ST} }}{(A)R}V_{i}$

## Comparative study

**Fig. 5:**
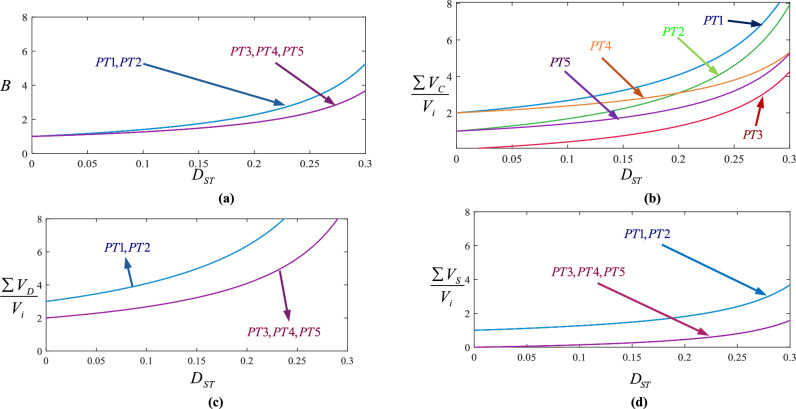
Comparison results between all proposed topologies; (**a**) boost factor; (**b**) total voltages across capacitors; (**c**) total blocked voltages across diodes; (**d**) blocked voltages across impedance side switch.

In this section, first, all proposed topologies are compared with each other individually to clearly identify their respective strengths and weaknesses. Then, all proposed topologies are thoroughly compared with several conventional topologies. The comparison results related to the proposed topologies are presented in Fig. 5. Figure 5a shows PT1 and PT2 have a better boost factor along with higher voltage stress across diodes and active switch. About the voltage stress across capacitors, Fig. 5b, it can be seen that PT3 and PT5 have lower voltage stress across capacitors. According to Figs. 5a and d, PT3, PT4 and PT5 have lower boost factor along with lower voltage stress across active switch. It is also worth noting that all the proposed topologies have the same number of components.

**Fig. 6:**
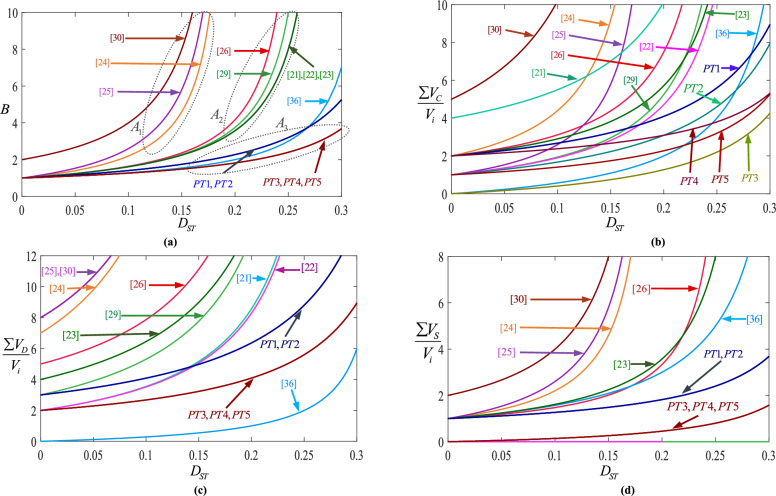
Comparison results among the proposed topologies and conventional ones; (**a**) boost factor; (**b**) total voltages across capacitors; (**c**) total blocked voltages across diodes; (**c**) total blocked voltages across impedance side switches.

For comparison purposes, the number of active and passive components, the boost-factor expressions, the total voltage stresses on capacitors, diodes, and active switches, the maximum efficiency values within the 100–250 W output-power range at ${D}_{ST}=0.24,$ and ${f}_{S}=10{\hspace{0.17em}kHz}$, and the maximum achievable voltage gain for all proposed and conventional topologies are compiled and summarized in Table 3. For clarity, the total voltage stresses of capacitors, active switches, and diodes are normalized with respect to the input voltage. In addition, because the efficiency depends on both the duty cycle and output load, the maximum efficiency obtained under identical operating conditions and within the 100–250 W power range is extracted for each converter and included in the table to ensure a fair comparison. Furthermore, the maximum boost factor corresponding to the maximum duty cycle for each topology is also reported. Figure 6 shows the comprehensive comparison for different points of view based on this table. According to Fig. 6a, at three different areas of duty cycles (A_1_, A_2_, and A_3_), there are rapid variations on the values of boost factors. This results that the boost factor has highly sensitive to duty cycles. In the first area (A_1_), which includes [24, 25] and [30], the sensitivity is very high, as a result, implementation in practical applications will be difficult. These topologies are not suitable for duty cycles above 0.15. In the second area (A_2_), which includes [21–23, 26] and [29], the duty cycle sensitivity is still high. But in the third area (A_3_), which includes [36] and all proposed topologies, it can be seen that the proposed topologies have a low sensitivity to the duty cycle, which can facilitate the implementation of these topologies. the problem with the topologies in the third area is that they have a lower boost factor than some conventional topologies. Figure 6b shows the total voltages across capacitors. According to this figure, the proposed topologies have low total voltages across capacitors. For example, at $D_{ST} = 0.2$, the normalized total voltages across capacitors for PT3, [36], PT5, PT4, PT2, PT1, [22, 23, 29], and [21] are equal to 1.27, 2, 2.27, 3.09, 3.09, 4.09, 4.71, 5.25, 5.71, and 10.14, respectively. By considering these results, the total voltage across capacitors of PT3 is 4.49 times lower than [23] and 7.98 times lower than [21]. For another example, at $D_{ST} = 0.15$, the normalized total voltages across capacitors for PT3, PT5, PT4, PT2, PT1, [23, 26] and [25] are equal to 0.74, 1.74, 2.66, 2.23, 3.23, 3.82, 4.37 and 5.80, respectively. By considering these results, the total voltage across capacitors of PT3 is 87.5% lower than [26] and 87.24% lower than [25]. Similarly, at $D_{ST} = 0.2$, the normalized total voltage across capacitors of PT5, PT4, PT2 and PT1 are 77.6%, 70%, 70%, 60% lower than [21], respectively.

**Table 3:** The comparative results for the proposed and conventional topologies.

	No. of L	No. of C	No. of D	No. of Active Switches	No. of Coupled Inductor	*B*	$\frac{{\sum {V_{C} } }}{{V_{i} }}$	$\frac{{\sum {V_{S} } }}{{V_{i} }}$	$\frac{{\sum {V_{D} } }}{{V_{i} }}$	Maximum efficiency values for output power between 100-250*W* at *D*_*ST*_ = 0.24 and *f*_*S*_ = 10*KHZ*	Maximum voltage gain
PT1	2	2	2	1	0	$\frac{1}{A}$	$\frac{{2 - D_{ST} }}{A}$	$\frac{{1 - D_{ST} }}{A}$	$\frac{{3 - D_{ST} }}{A}$	93.14% for 147.51*W*	*D*_*ST*_ = 0.38 *B* = 227
PT2	2	2	2	1	0	$\frac{1}{A}$	$\frac{{2D_{ST} - D_{ST}^{2} + 1}}{A}$	$\frac{{1 - D_{ST} }}{A}$	$\frac{{3 - D_{ST} }}{A}$	91.02% for 162.07*W*	*D*_*ST*_ = 0.38 *B* = 227
PT3	2	2	2	1	0	$\frac{{1 - D_{ST} }}{A}$	$\frac{{3D_{ST} - D_{ST}^{2} }}{A}$	$\frac{{D_{ST} }}{A}$	$\frac{{2 - D_{ST} }}{A}$	89.65% for 103.76*W*	*D*_*ST*_ = 0.38 *B* = 141
PT4	2	2	2	1	0	$\frac{{1 - D_{ST} }}{A}$	$\frac{{2 - 3D_{ST} - D_{ST}^{2} }}{A}$	$\frac{{D_{ST} }}{A}$	$\frac{{2 - D_{ST} }}{A}$	94.21% for 103.76*W*	*D*_*ST*_ = 0.38 *B* = 141
PT5	2	2	2	1	0	$\frac{{1 - D_{ST} }}{A}$	$\frac{1}{A}$	$\frac{{D_{ST} }}{A}$	$\frac{{2 - D_{ST} }}{A}$	92.52% for 103.76*W*	*D*_*ST*_ = 0.38 *B* = 141
[21]	4	4	5	0	0	$\frac{1}{{2D_{ST}^{2} - 4D_{ST} + 1}}$	$\frac{{4 - 6D_{ST} + D_{ST}^{2} }}{{2D_{ST}^{2} - 4D_{ST} + 1}}$	0	$\frac{{2 + 2D_{ST} }}{{2D_{ST}^{2} - 4D_{ST} + 1}}$	–	*D*_*ST*_ = 0.29 *B* = 122
[22]	4	4	5	0	0	$\frac{1}{{2D_{ST}^{2} - 4D_{ST} + 1}}$	$\frac{{1 + 2D_{ST} - 2D_{ST}^{2} }}{{2D_{ST}^{2} - 4D_{ST} + 1}}$	0	$\frac{{2 - 2D_{ST}^{2} + 2D_{ST} }}{{2D_{ST}^{2} - 4D_{ST} + 1}}$	–	*D*_*ST*_ = 0.29 *B* = 122
[23]	2	2	4	1	0	$\frac{1}{{2D_{ST}^{2} - 4D_{ST} + 1}}$	$\frac{{2 - 2D_{ST} }}{{2D_{ST}^{2} - 4D_{ST} + 1}}$	$\frac{1}{{2D_{ST}^{2} - 4D_{ST} + 1}}$	$\frac{4}{{2D_{ST}^{2} - 4D_{ST} + 1}}$	–	*D*_*ST*_ = 0.29 *B* = 122
[24]	2	2	7	2	0	$\frac{{1 + D_{ST} }}{{1 - 5D_{ST} }}$	$\frac{{2 + 2D_{ST} }}{{1 - 5D_{ST} }}$	$\frac{{1 + D_{ST} }}{{1 - 5D_{ST} }}$	$\frac{{7 + 7D_{ST} }}{{1 - 5D_{ST} }}$	93.48% for 108.91*W*	*D*_*ST*_ = 0.19 *B* = 23.8
[25]	0	1	2	1	1*	$\frac{{1 + 3D_{ST} }}{{1 - 5D_{ST} }}$	$\frac{{1 + 3D_{ST} }}{{1 - 5D_{ST} }}$	$\frac{{1 + 3D_{ST} }}{{1 - 5D_{ST} }}$	$\frac{8}{{1 - 5D_{ST} }}$	–	*D*_*ST*_ = 0.19 *B* = 31.4
[26]	3	2	5	1	0	$\frac{1}{{1 - 4D_{ST} + D_{ST}^{2} }}$	$\frac{{2 - D_{ST} }}{{1 - 4D_{ST} + D_{ST}^{2} }}$	$\frac{{1 - D_{ST} }}{{1 - 4D_{ST} + D_{ST}^{2} }}$	$\frac{{5 - 2D_{ST} }}{{1 - 4D_{ST} + D_{ST}^{2} }}$	90.92% for 112.57*W*	*D*_*ST*_ = 0.26 *B* = 36.23
[29]	4	4	5	0	0	$\frac{{1 + D_{ST} }}{{1 - 3D_{ST} - 2D_{ST}^{2} }}$	$\frac{{(1 + D_{ST} )(1 + 2D_{ST} )}}{{1 - 3D_{ST} - 2D_{ST}^{2} }}$	0	$\frac{{3 + 6D_{ST} + D_{ST}^{2} }}{{1 - 3D_{ST} - 2D_{ST}^{2} }}$	88.2% for 111.87*W*	*D*_*ST*_ = 0.28 *B* = 400
[30]	3	4	5	1	0	$\frac{2}{{1 - 5D_{ST} }}$	$\frac{{5 + D_{ST} }}{{1 - 5D_{ST} }}$	$\frac{2}{{1 - 5D_{ST} }}$	$\frac{8}{{1 - 5D_{ST} }}$	87.97% for 116.67*W*	*D*_*ST*_ = 0.19 *B* = 40
[36]	2	2	3	2	0	$\frac{{1 - D_{ST} }}{{1 - 3D_{ST} }}$	$\frac{{4D_{ST} }}{{1 - 3D_{ST} }}$	$\frac{{1 + D_{ST} }}{{1 - 3D_{ST} }}$	$\frac{{2D_{ST} }}{{1 - 3D_{ST} }}$	90.16% for 111.23*W*	*D*_*ST*_ = 0.33 *B* = 67

*The ratio of turns is assumed 1.

At $D_{ST} = 0.15$, the total voltage across capacitors of PT5, PT4, PT2 and PT1 are 60%, 39%, 49%, 27% lower than [26], respectively. Figure 6c shows normalized total blocked voltages across diodes. According to this figure, the proposed topologies have low total voltages across diodes. For example, at $D_{ST} = 0.2$, the normalized total blocked voltages across diodes for [36], PT3, PT4, PT5, PT1, PT2, [22], and [21] are equal to 1, 4.09, 4.09, 4.09, 6.36, 6.36, 8.29, and 8.57, respectively. By considering these results, the normalized total blocked voltages across diodes of [36] is the lowest value and after that, the proposed topologies have lower normalized total blocked voltages across diodes than others. It can be concluded that PT3, PT4 and, PT5 are 53.5% lower than [21] and 51.8% lower than [22]. For $D_{ST} = 0.15$, the total blocked voltages across diodes for [36], PT3, PT4, PT5, PT1, PT2, [21–23, 29], and [26] are equal to 0.54, 3.23, 3.23, 3.23 4.97, 4.97, 5.06, 5.16, 7.76, 8.98, and 11.12, respectively. By considering these results, the normalized total voltage across diodes of PT3, PT4 and PT5 are 64.23% lower than [23] and 71.16% lower than [26]. Furthermore, at $D_{ST} = 0.2$, the normalized total blocked voltages across diodes for PT1, PT2, PT3, PT4, and PT5 are 26%, 26%, 53%, 53%, and 53% lower than [21], respectively. Figure 6d shows the normalized total blocked voltages across active switches. According to this figure, the proposed topologies have small total blocked voltages across active switches. For example, at $D_{ST} = 0.2$, the total blocked voltages across active switches for PT3, PT4, PT5, PT1, PT2, [26, 36], and [23] are equal to 0.45, 0.45, 0.45, 1.82, 1.82, 3, 3.33, and 3.57, respectively.

**Fig. 7:**
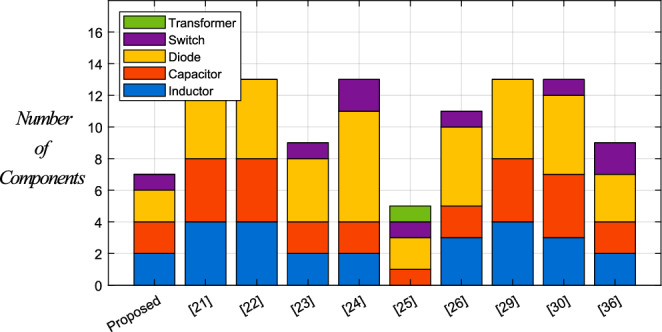
Comparison of number of components.

By considering these results, the total voltage across active switches of PT3, PT4, and PT5 are 86% lower than [26], and 87% lower than [23]. For $D_{ST} = 0.15$, the total blocked voltages across active switches for PT3, PT4, PT5, PT1, PT2, [23, 26, 36] and [24] are equal to 0.26, 0.26, 0.26, 1.48, 1.48, 2.1, 2.01, 2.25 and 4.6, respectively. According to these results, for $D_{ST} = 0.15$, PT3, PT4, PT5, PT1, and PT2 are 94%, 94%, 94%, 68%, and 68% lower than [24]. Figure 7 shows the bar chart of number of passive and active components. According to this figure, the proposed topologies use fewer number of elements.

**Fig. 8:**
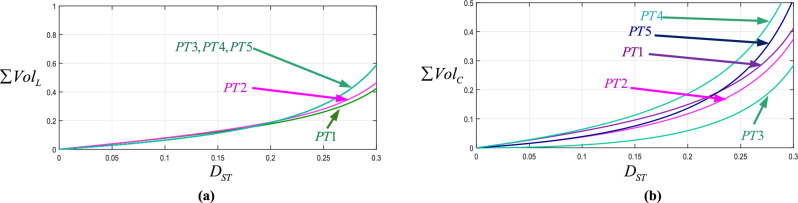
Total volumetric comparison of proposed topologies; (**a**) inductors; (**b**) capacitors.

**Fig. 9:**
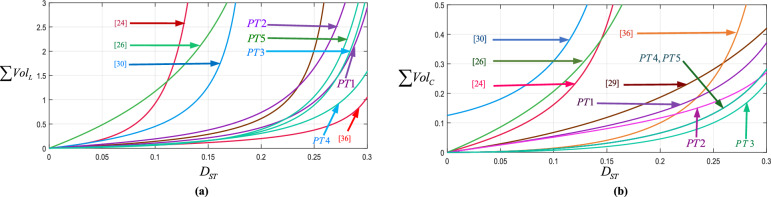
Total volumetric comparison of proposed topologies and some conventional topologies; (**a**) inductors; (**b**) capacitors.

The extraction of the power density of the proposed and conventional topologies is an effective way to evaluate their quality. The power density is influenced by calculating the volumes of their inductors and capacitors. The volume of the inductor, which is related to stored energy ($W_{L} = 1/2LI^{2}$), is divided by the inductor’s volumetric energy density (*ρ*_*E,L*_). Additionally, the volume of the capacitor is determined by dividing its stored energy ($W_{C} = 1/2CV^{2}$) by its volumetric energy density *(ρ*_*E,C*_). In the following, first, the total inductors and capacitors’ volumes (*Vol*_*inductors*_, *Vol*_*capacitor*_) of PT1 are calculated as (38) and (39), respectively. Then, the overall volume metric (*Vol*_*converter*_) is the sum of Eqs. (38) and (39), which can be written as (40). Finally, the overall volume metric (*Vol*_*converter*_) of proposed and conventional topologies is summarized in Table 4. Figure 8 shows variations of volume versus the duty cycle of proposed topologies individually and Fig. 9 shows variations in volume versus the duty cycle of proposed topologies and conventional topologies. Based on Fig. 8a and b, PT1 and PT2 have the highest capacitors volume and the lowest inductors volume values among the proposed topologies. Also, PT3 has the lowest capacitors volume among them.

**Table 4:** Parameters of volume comparative study.

References	Passive components volume $/T_{S} P_{o}$
PT1	$\frac{{(D_{ST}^{2} - 3D_{ST} + 3)D_{ST} (1 - D_{ST} )^{2} }}{{4(D_{ST}^{2} - 3D + 1)x\% \rho_{E,L} }} + \frac{{0.25D_{ST} (1 - D_{ST} )(D_{ST}^{2} - 2D + 2)}}{{(D_{ST}^{2} - 3D + 1)y\% \rho_{E,C} }}$
PT2	$\frac{{D_{ST} (1 - D_{ST} )^{2} (2D_{ST} - 3)}}{{4(D_{ST}^{2} - 3D + 1)x\% \rho_{E,L} }} + \frac{{0.5D_{ST} (D_{ST} - 1)}}{{(D_{ST}^{2} - 3D_{ST} + 1)y\% \rho_{E,C} }}$
PT3	$\frac{{D_{ST} (D_{ST}^{2} - 2D + 2)}}{{4(D_{ST}^{2} - 3D + 1)x\% \rho_{E,L} }} + \frac{{(2D_{ST} - 3)D_{ST}^{2} }}{{4(D_{ST}^{2} - 3D_{ST} + 1)(1 - D_{ST} )y\% \rho_{E,C} }}$
PT4	$\frac{{(D_{ST}^{2} - 2D_{ST} + 2)D_{ST} }}{{4(D_{ST}^{2} - 3D + 1)x\% \rho_{E,L} }} + \frac{{D_{ST} (D_{ST}^{2} - 2D_{ST} + 2)}}{{4(D_{ST}^{2} - 3D_{ST} + 1)y\% \rho_{E,C} }}$
PT5	$\frac{{D_{ST} (D_{ST}^{2} - 2D_{ST} + 2)}}{{4(D_{ST}^{2} - 3D + 1)x\% \rho_{E,L} }} + \frac{{(D_{ST} + 1)D_{ST} }}{{4(D_{ST}^{2} - 3D_{ST} + 1)y\% \rho_{E,C} }}$
[24]	$$\frac{{2D_{ST} (1 + D_{ST} )(1 - 2D_{ST} )(1 - D_{ST} )^{4} }}{{(1 - 5D_{ST} )^{3} x\% \rho_{E,L} }} + \frac{{D_{ST} (1 - D_{ST} )^{2} }}{{(1 - 5D_{ST} )y\% \rho_{E,C} }}$$
[26]	$$\frac{{(17D_{ST}^{2} - 36D_{ST} + 67)(1 - D_{ST} )D_{ST} }}{{8(D_{ST}^{2} - 4D_{ST} + 1)x\% \rho_{E,L} }} + \frac{{( - 0.5D_{ST}^{2} - 1.5 + D_{ST} )(D_{ST} - 1)D_{ST} }}{{(D_{ST}^{2} - 4D_{ST} + 1)y\% \rho_{E,C} }}$$
[29]	$$\frac{{D_{ST} (1 - D_{ST} )^{2} (2 + D_{ST} )}}{{4(1 + 4D)(D_{ST} - 0.5)(1 - 3D_{ST} - 2D_{ST}^{2} )x\% \rho_{E,L} }} + \frac{{(0.5 + D_{ST} )(D_{ST} - 1)D_{ST} }}{{(2D_{ST} - 1)y\% \rho_{E,C} }}$$
[30]	$$\frac{{5D_{ST} (D_{ST} - 1)}}{{2(5D_{ST} - 1)x\% \rho_{E,L} }} + \frac{{14D_{ST}^{2} + D_{ST} + 1}}{{8(1 - 5D_{ST} )y\% \rho_{E,C} }}$$
[36]	$$\frac{{D_{ST} (1 - D_{ST} )}}{{2(1 - 3D_{ST} )x\% \rho_{E,L} }} + \frac{{D_{ST}^{2} (D_{ST}^{2} - 2D_{ST} + 1)}}{{(1 - D_{ST} )^{2} (1 - 3D_{ST} )y\% \rho_{E,C} }}$$

Based on Fig. 9a the proposed topologies and [36] demonstrate low volume of inductors for different duty cycles. As can be seen in this figure, [36] has lower inductors volume than others. It is worth noting that [36] has higher total blocked voltages across active switches and lower boost factor than proposed topologies. Also, in [36] one extra diode and one extra active switch compared to proposed topologies are used. In Fig. 9b, it can be concluded that PT2, PT3, and PT4 have smaller capacitor volume (Table 5).

38 $$ Vol_{inductors} = \frac{{\sum {E_{Li} } }}{{\rho_{E,L} }} = \frac{{(D_{ST}^{2} - 3D_{ST} + 3)D_{ST} (1 - D_{ST} )^{2} }}{{4(D_{ST}^{2} - 3D + 1)x\% \rho_{E,L} }} $$

39 $$ Vol_{capacitors} = \frac{{\sum {E_{Ci} } }}{{\rho_{E,C} }} = \frac{{0.25D_{ST} (1 - D_{ST} )(D_{ST}^{2} - 2D + 2)P_{o} }}{{(D_{ST}^{2} - 3D + 1)y\% f_{s} \rho_{E,C} }} $$

40 $$ Vol_{converter} = \left[ {\frac{{(D_{ST}^{2} - 3D_{ST} + 3)D_{ST} (1 - D_{ST} )^{2} }}{{4(D_{ST}^{2} - 3D + 1)x\% \rho_{E,L} }} + \frac{{0.25D_{ST} (1 - D_{ST} )(D_{ST}^{2} - 2D + 2)}}{{(D_{ST}^{2} - 3D + 1)y\% \rho_{E,C} }}} \right]\frac{{P_{o} }}{{f_{s} }} $$

**Table 5:** Design parameters.

*f*_*S*_	10kHz
*R*_*L*_	100Ω
*D*_*ST*_	0.24
*C*_1_, *C*_2_	22µF
*L*_1_, *L*_2_	0.6mH
50*V*	*V*_i_

**Fig. 10:**
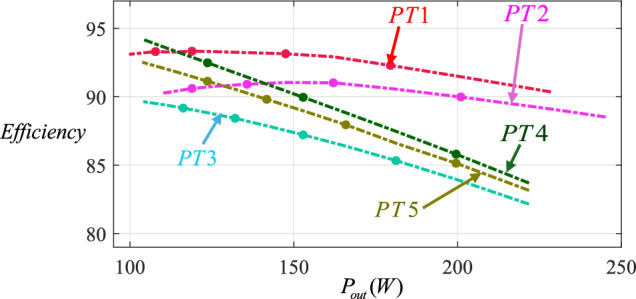
Comparison of efficiency between all proposed topologies versus output power.

**Fig. 11:**
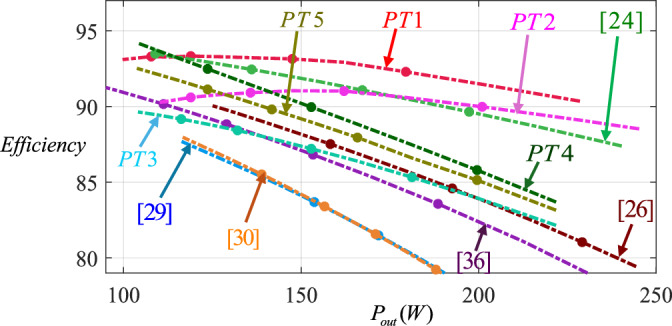
Comparison of efficiency among the proposed topologies and conventional ones versus output power.

The efficiency diagrams of the proposed topologies versus output power (*P*_*out*_) individually and the proposed and conventional topologies versus output power (*P*_*out*_) are provided in Figs. 10 and 11, respectively. According to Fig. 10, PT1 has better efficiency than the other proposed topologies and PT3 has lower efficiency than the others. Also, this figure evaluates the performance of the proposed topologies.

Furthermore, Fig. 10 shows that the efficiency of PT1 is higher than the efficiency of other proposed and conventional topologies. For example, for $P_{out} = 200W$, PT1 (by efficiency near 92%), PT2 (by efficiency near 90%), [24] (by efficiency near 88.5%), PT4 (by efficiency near 86%), PT5 (by efficiency near 85.2%), PT3 (by efficiency near 84%), [26] (by efficiency near 84%) and [36] (by efficiency near 82%), have efficiencies from highest to lowest. Ref. [24] has a better efficiency than PT4, PT5, and PT3. But it needs a high number of components along with high blocking voltages across switches and diodes and large volume of inductors and capacitors. So, it can be concluded that the proposed topologies have acceptable efficiency.

These figures were obtained by simulating the proposed topology and the comparable existing topologies under exactly the same conditions and using the same control method. First, all the proposed and previous topologies were simulated in PSCAD software in real conditions, and identical values for the diode series resistance (*r*_*D*_ = 0.1*Ω*), switch series resistance (*r*_*S*_ = 0.15*Ω*), as well as the equivalent resistance of inductors (*r*_*L*_ = 0.4*Ω*) and the equivalent resistance of capacitors (*r*_*C*_ = 0.012*Ω*) were assigned to each topology separately. Then, the efficiencies of the topologies were evaluated at different output power levels under identical operating conditions. Finally, the efficiency comparison figure for the all topologies was generated.

## Dynamic modelling and voltage loop controller design

This section presents the small-signal modeling and closed-loop voltage control design of the proposed topology, accompanied by the corresponding Bode diagrams.

###  Small signal modelling

The state-space averaging method is applied to model the proposed converter over an entire switching cycle. First, the state-space equations for the two time intervals, *D*_*ST*_*T*_*s*_ and (1*–D*_*ST*_)*T*_*s*_, are written, and then they are averaged using a matrix to approximate the overall behavior of the converter. Consequently, the averaged state-space matrix of the proposed converter is expressed as follows:

41 $$ \left[ \begin{gathered} \frac{{di_{L1} }}{dt} \hfill \\ \frac{{di_{L2} }}{dt} \hfill \\ \frac{{dv_{C1} }}{dt} \hfill \\ \frac{{dv_{C2} }}{dt} \hfill \\ \end{gathered} \right] = \left[ {\begin{array}{*{20}c} 0 & 0 & {\frac{1}{{L_{1} }}} & { - \frac{(1 - d)}{{L_{1} }}} \\ 0 & 0 & { - \frac{(1 - d)}{{L_{1} }}} & {\frac{d}{{L_{2} }}} \\ { - \frac{1}{{C_{1} }}} & {\frac{(1 - d)}{{C_{1} }}} & 0 & 0 \\ {\frac{(1 - d)}{{C_{2} }}} & { - \frac{d}{{C_{2} }}} & 0 & { - \frac{(1 - d)}{{RC_{2} }}} \\ \end{array} } \right]\left[ {\begin{array}{*{20}c} {i_{L1} } \\ {i_{L2} } \\ {v_{C1} } \\ {v_{C2} } \\ \end{array} } \right] + \left[ {\begin{array}{*{20}c} 0 \\ {\frac{{v_{i} }}{{L_{2} }}} \\ 0 \\ 0 \\ \end{array} } \right] $$

42 $$ V_{o} = \left[ {\begin{array}{*{20}c} 0 & 0 & 0 & 1 \\ \end{array} } \right]\left[ {\begin{array}{*{20}c} {i_{L1} } \\ {i_{L1} } \\ {v_{C1} } \\ {v_{C2} } \\ \end{array} } \right] $$

In the above relationships, "*d*" represents the duty cycle at time *t*. The general forms of relationships (41) and (42) are defined as:

43 $$ \left\{ \begin{gathered} x^{\prime} = Ax + BU \hfill \\ y = Cx \hfill \\ \end{gathered} \right. $$

Taking the Laplace transform of these relationships yields the converter’s transfer function as:

44 $$ G(s) = \frac{Y(s)}{{U(s)}} = C(sI - A)^{ - 1} \,B $$

Using relationships (41), (42), and (43), and assuming *d* = *D*, the transfer function of the proposed converter is obtained as:

45 $$ G(s) = \frac{ - DR}{{C_{2} L_{2} Rs^{2} + L_{2} (1 - D)s + D^{2} R}} $$

###  Closed-loop voltage controller

The proposed inverters use five switches, with the output voltage controlled by adjusting the duty cycle of the switches via Pulse Width Modulation (PWM). To maintain a constant output voltage, even with input voltage variations, a PI (Proportional-Integral) controller is employed.

**Fig. 12:**
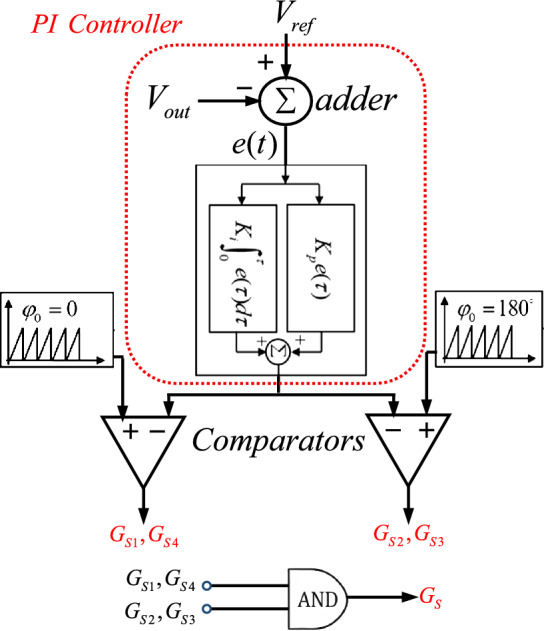
Control block diagram of the proposed inverters using PI Controller and PWM.

As shown in Fig. 12, the control system includes four main components: an adder (or error detector), a PI (Proportional-Integral) controller, a carrier waveform, and a comparator. The system works by first sampling the output voltage and comparing it with a reference voltage (*V*_*ref*_) using the adder. The error signal (e(t)) from the adder is sent to the PI controller.

The PI controller combines proportional action, which adjusts based on the current error, and integral action, which accounts for accumulated past errors. This ensures both immediate and long-term correction of the output voltage. The controller’s output is compared with a carrier waveform in the comparator, generating a PWM signal that regulates the switches *S*_1_, *S*_2_, *S*_3_ and, *S*_4_.

Finally, the comparator’s output is used to generate the control signal *S* through an AND gate, stabilizing the output voltage at the desired value. This feedback loop ensures the inverter’s output voltage remains constant despite variations in the input.

To design an appropriate PI controller for the proposed converter, the proportional and integral coefficients were determined using MATLAB’s PID Tuning feature. The calculated coefficients are as follows:

46 $$ G_{PI} (s) = k_{p} + \frac{{k_{i} }}{s} $$

**Fig. 13:**
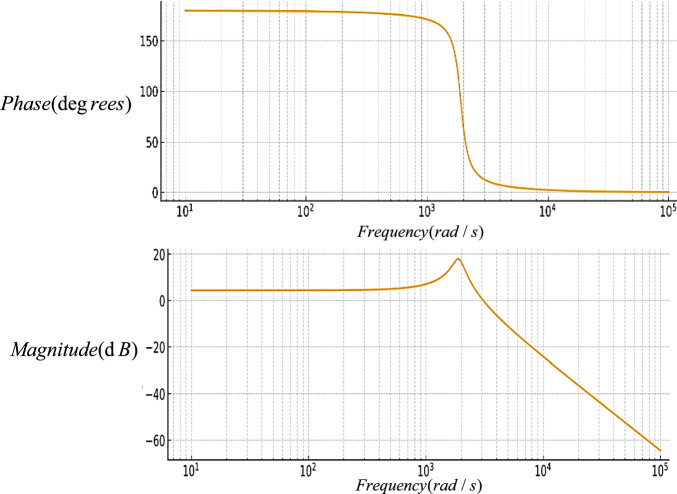
The bode diagram of the proposed converter before compensation.

The Bode diagram of the proposed converter before compensation is shown in Fig. 13. According to this figure, the system gain remains constant and negative at low frequencies, while it decreases with a slope of approximately − 40 *dB/dec* at higher frequencies. This behavior is attributed to the presence of two dominant poles in the system’s transfer function. Moreover, the phase response approaches − 180° in the critical frequency region. To improve system stability and enhance the low-frequency gain, a proportional–integral (PI) controller was designed with the following relationship:

47 $$ G_{PI} (s) = \frac{6.2s + 1}{{s + 0.055044}} $$

**Fig. 14:**
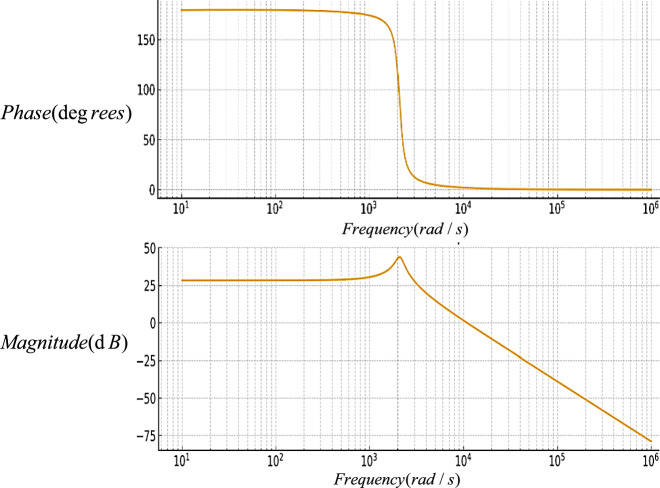
The bode diagram of the proposed converter after compensation.

In Fig. 14, the Bode diagram of the proposed converter is drawn after compensation. According to this figure, a noticeable improvement in phase margin is observed, confirming the system’s suitability for stable closed-loop operation. The designed PI controller enhances the transient performance, improves output voltage regulation accuracy, and reduces the system’s sensitivity to parameter variations and load disturbances. Overall, the comparison between these two plots (Figs. 13 and 14), confirms the effectiveness of the proposed compensation strategy and validates the effectiveness of the control design in terms of dynamic performance and stability assurance.

## Experimental results

**Fig. 15:**
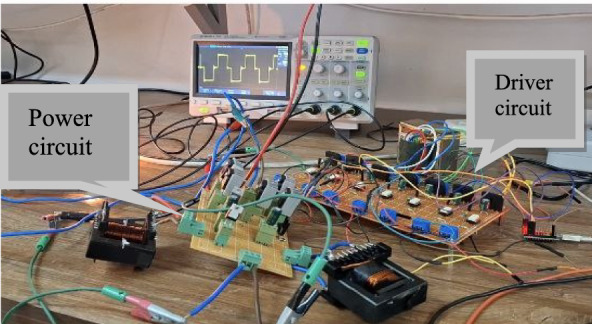
Prototype of PT1.

In this section, the experimental results of PT1 are given to reconfirm the accuracy of theoretical analyses. First, the appropriate values of inductors and capacitors should be calculated. According to (34)–(37) and by considering inductors’ current ripple (*x*_*L*_%) equal to 0.5 and capacitors’ voltage ripple (*x*_*C*_%) equal to 0.05, switching frequency (*f*_*S*_) equal to 10kHz, load output resistance (*R*_*L*_) equal to 100Ω, and *D*_*ST*_ = 0.24, the amounts of both capacitors (*C*_*1*_, *C*_*2*_) and both inductors (*L*_*1*_, *L*_*2*_) will be equal to 22µF and 0.6mH, respectively. The amount of input voltage is considered 50*V*. It is also notable that the used control method is similar to Fig. 2 in which of *f*_*car*_ = 10kHz, *f*_*ref*_ = 50Hz. In the prototype, IRFP460 and MUR1560 are used for switches and rectifier diode, respectively. The design parameters are summarized in Table. 5. The prototype of PT1 is shown in Fig. 15. In the prototype, the implementation of the control method used in the experiment begins by calculating the required gate pulse width for each power switch. After determining these values, an Arduino Pro Micro microcontroller is programmed to generate the corresponding gate signals. However, since the microcontroller’s output voltage (maximum 4.5 *V*) is insufficient to drive the MOSFETs directly, a gate driver circuit (TLP250) is employed. This driver boosts the signal voltage to approximately 9 *V*, providing the necessary level to reliably turn the MOSFETs on and off. Additionally, a two-channel digital oscilloscope (model GPS-1102B) with 10*MHz* bandwidth and 1*GSa/s* sampling rate along with isolated high voltage probe (Hantek HT8100) and a power analyzer (model C.A 8336) were used in this experiment.

**Fig. 16:**
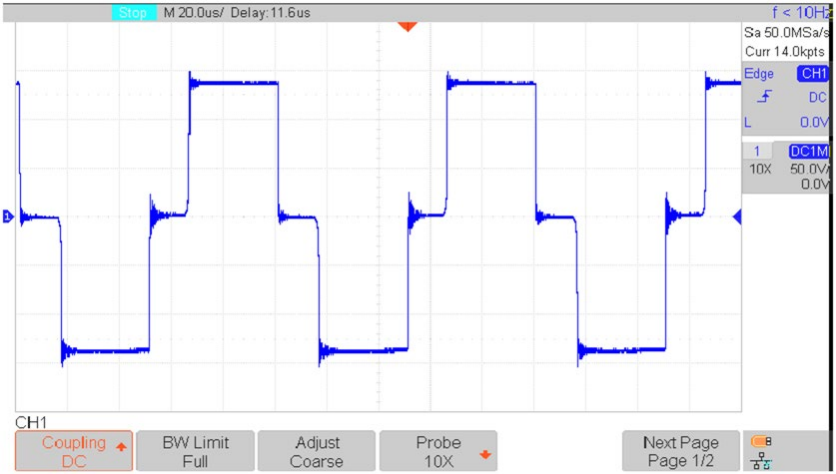
Experimental result for output voltage.

**Fig. 17:**
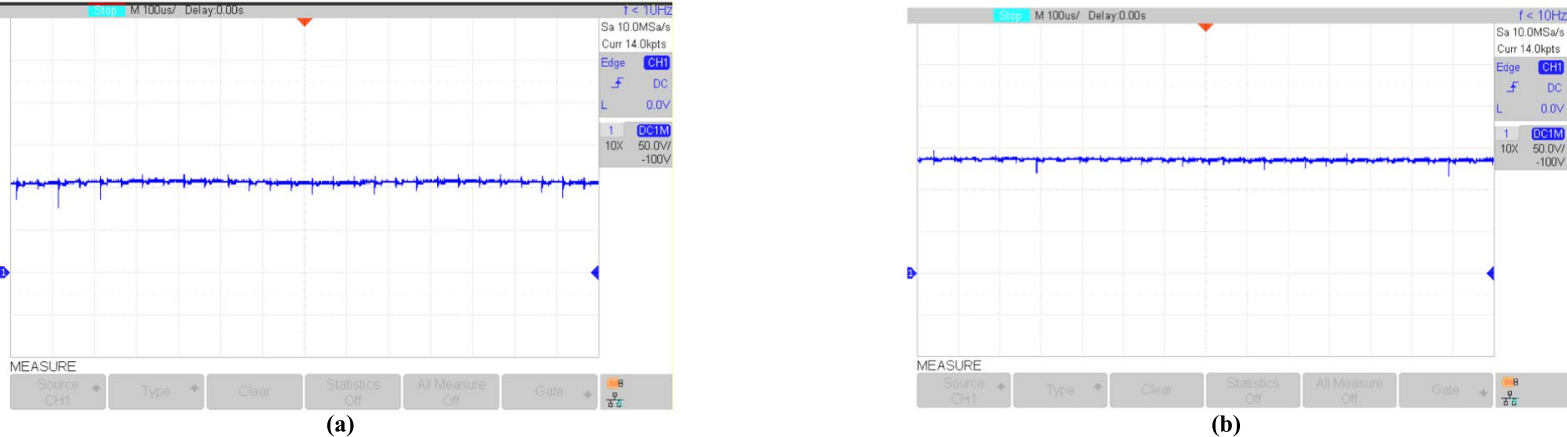
Experimental results; (**a**) voltages across *C*_*1*_; (**b**) voltages across *C*_*2*_.

**Fig. 18:**
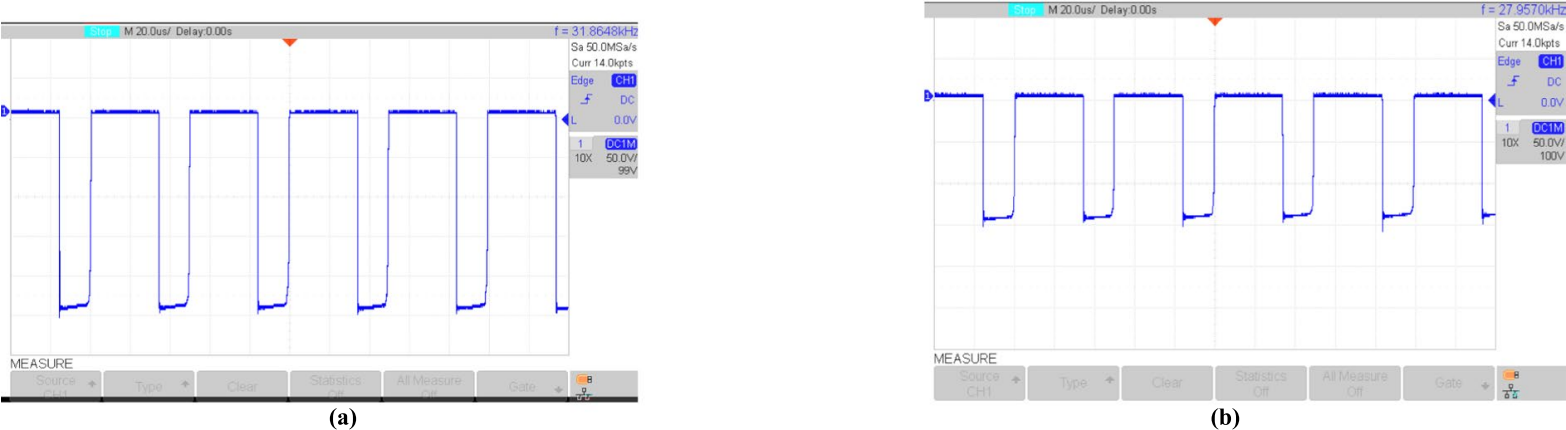
Experimental results; (**a**) voltage on *D*_*1*_; (**b**) voltage on *D*_*2*_.

**Fig. 19:**
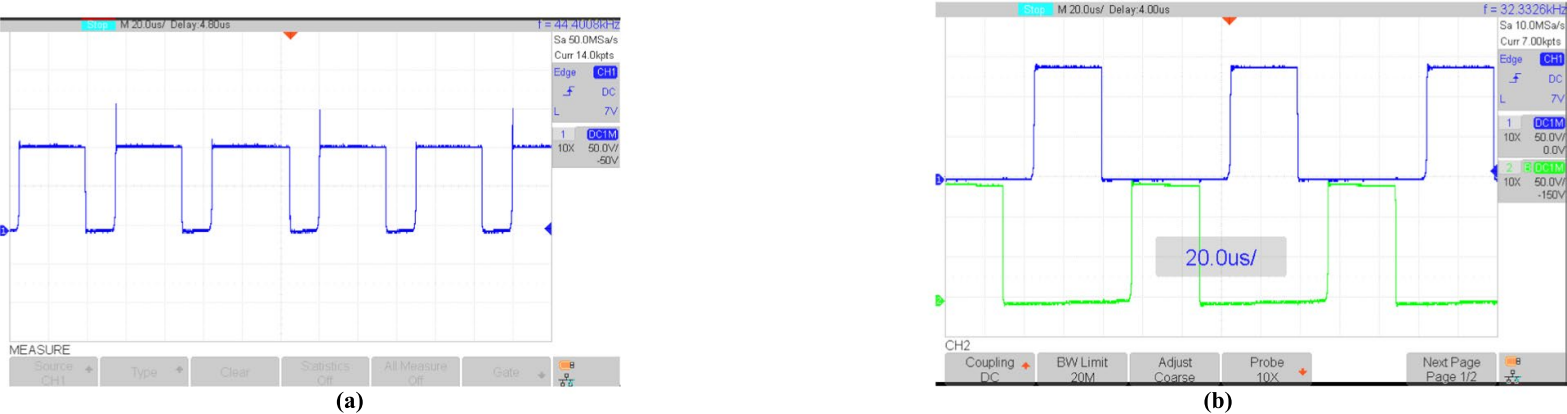
Experimental results; (**a**) voltage across *S*; (**b**) voltage across *S*_*1*_, *S*_*2*_, *S*_*3*_, *S*_*4*_.

**Fig. 20:**
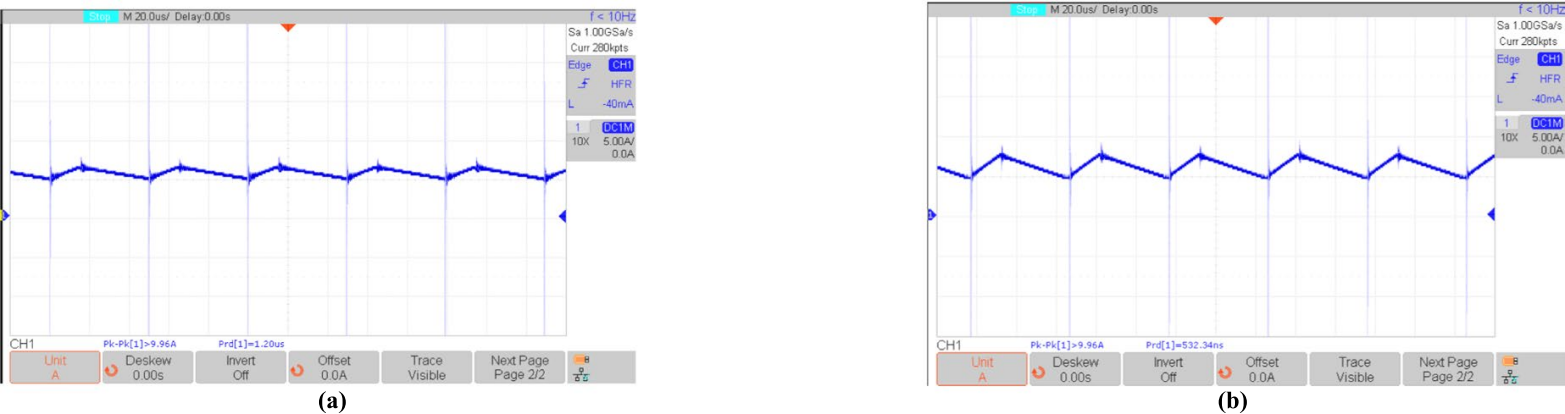
Experimental results; (**a**) current through *L*_*1*_; (**b**) current through *L*_*2*_.

**Fig. 21:**
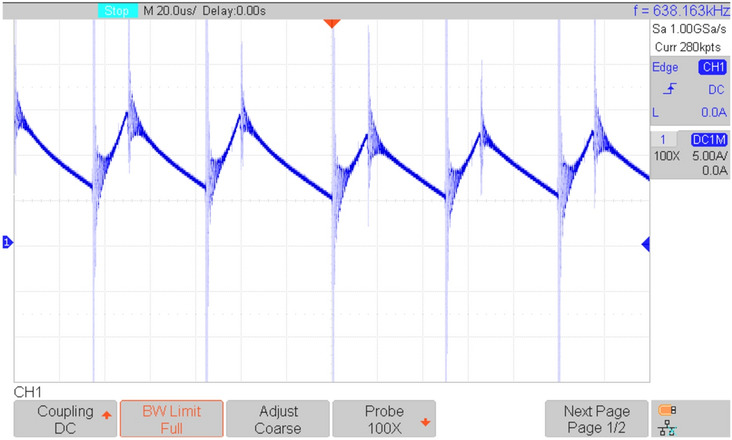
Experimental result for input current in R = 100 Ω.

Figures 16, 17, 18, 19, 20 and 21 show the experimental results for key waveforms. Figure 16 demonstrates the output voltage waveform. As seen in this figure, the magnitude of the output voltage is approximately 140V. According to (20), the output voltage is 148 V. Comparing the theoretical and experimental results shows that there is good agreement between them. Considering this point in theoretical analysis, all elements are assumed to be ideal, thus, a small difference exists between the theoretical and experimental results. Equation (20) predicts that duty cycle variations affect both amplitude and width of the output waveform. Moreover, the output voltage waveform is a three-level waveform with a frequency equal to that of the control method. According to (17) and (18), the voltages of capacitors *C*_*1*_ and *C*_*2*_ are equal to 112.55V and 148.10V, respectively. By comparing these values with Fig. 17a and b, it can be seen that there is a slight difference between them. Moreover, the capacitor voltage waveforms in these figures are continuous and stable, which contributes to improved system stability, reduced voltage stress on the switches, and minimized output voltage ripple and enhancing overall efficiency.

By considering (1), (2), and (6), the voltages of diodes *D*_*1*_ and *D*_*2*_ are equal to 260.67*V* and 148*V*, respectively. From Fig. 18a and b, the diode voltages in the experimental results are near 246V and 140V, respectively. Comparing these values with theoretical results shows that there is a good agreement between them. Also, these figures confirm the ST and nST states of diodes. Figure 19a and b show the voltage stress on switches *S*, *S*_*1*_, *S*_*2*_, *S*_*3,*_ and *S*_*4*_. In theoretical analysis, the voltage stress of switch *S* is 112.55V, which is confirmed by Fig. 19a. Also, according to Fig. 19b, the absolute value of the maximum voltage across H-bridge switches is equal to the output voltage. In theoretical analyses, the average values of the inductors’ current of *L*_1_ and *L*_2_ are equal to 5.06A and 6.66A, respectively, and these values are reconfirmed by Figs. 20a and b. Also, these figures show that the inductor currents do not reach zero during the switching cycle. This results in smoother current transitions, reduced switching losses, and improved system stability and efficiency.

Figure 21 shows the input current of the proposed topology. According to this figure, it can be seen that the input current is continuous.

**Fig. 22:**
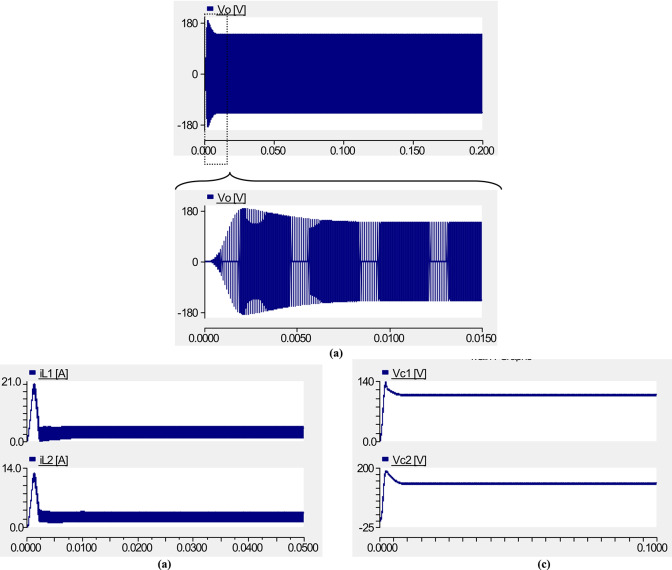
Simulation results for transient state; (**a**) output voltage; (**b**) current through inductors; (**c**) voltage across capacitors.

The simulation results for the transient state of PT1 are shown in Fig. 22. As can be seen in this figure, the overshoot voltage of boost-factor, the overshoot current of inductors *L*_1_ and *L*_2_ and the over shoot voltage of capacitors *C*_1_ and *C*_2_ are close to 190*V*, 21*A*, 14*A* and 140*V*, 180*V*, respectively. It is worth mentioning that the simulation results for transient state are given for the real condition of elements.

Considering the application of the presented topologies, it’s suggested that the proposed topologies are specifically designed for non-grid-connected industrial applications that require high-frequency, non-sinusoidal output voltages, such as:

1. Electrochemical processes, including electroplating, where a controllable high-frequency pulsed voltage can enhance reaction efficiency, improve deposition quality, and reduce electrode wear.
2. Induction heating systems, which rely on high-frequency voltage waveforms to generate localized heating through induced eddy currents, and where sinusoidal waveforms are not a necessity.

These types of applications do not require sinusoidal voltage outputs or compliance with grid-related harmonic standards. Instead, they benefit from high switching frequency, adjustable output amplitude, and efficient power delivery, all of which are offered by the proposed inverter topology. Also, it is worth mentioning that Induction heating systems do not require a common ground between input and output.

In these cases, it is essential to generate output voltages with variable amplitude and frequency. As demonstrated by Table. 2, the boost factor of the proposed converters can be effectively controlled by adjusting the *D*_*ST*_. This feature allows flexible regulation of the output voltage level, enabling compatibility with different process requirements.

**Fig. 23:**
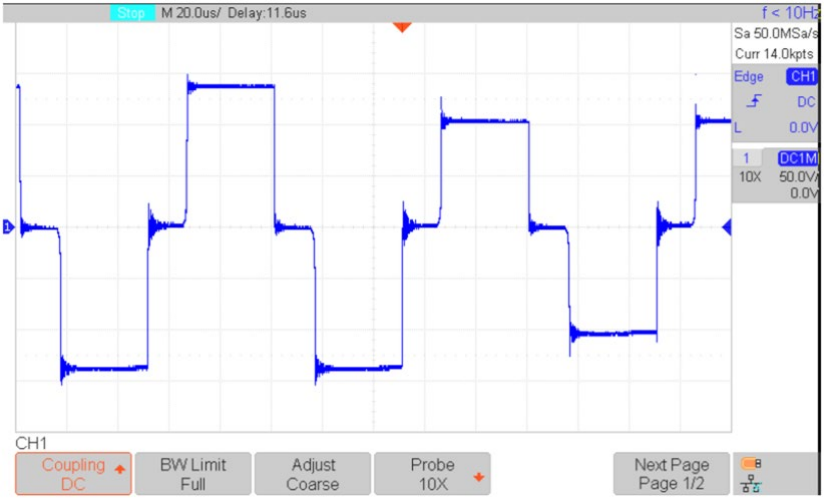
Output voltage response to *D*_*ST*_ adjustment.

This controllability is illustrated in Fig. 23, which shows the experimental output voltage waveform of the first proposed topology for different values of *D*_*ST*_. Initially, *D*_*ST*_ = 0.24 is applied for two switching periods. Then, the duty cycle is reduced to 0.20 for the next two periods. As observed, the output voltage amplitude changes accordingly, demonstrating the practical ability of the topology to regulate the output in real time.

Such characteristics make the proposed inverter well-suited for industrial applications where precise control of high-frequency voltage waveforms is required, eliminating the need for conventional output filters.

## Proposed topology’s losses

In This section, the calculation of losses of all component of first proposed topology to identify which components contribute the most to the overall losses were done. It should also be noted that the loss calculations provided in the following subsections are applicable to all five topologies. Due to the inherent series resistances and their corresponding voltage drops, all components exhibit measurable power losses. To quantify these losses, the following assumptions are considered:

- Losses in passive components, including inductors and capacitors, are represented using their equivalent series resistances, denoted as *r*_*L*_ for inductors and *r*_*c*_ for capacitors.
- All diodes and switches are assumed to have identical characteristics.
- The inductor current ripple is neglected.
- Active components, namely switches and diodes, incur two types of losses: conduction losses and switching losses. Conduction losses occur when the device is in the ON state, causing a voltage drop across its series resistance. Switching losses arise during transitions between ON and OFF states. For diodes, the series resistance and forward voltage drop are represented by *r*_*D*_ and *V*_*FD*_ respectively. For switches, these parameters are denoted as *r*_*S*_ and *V*_*FS*_.

###  Switches losses

As shown in Fig. 2, the proposed topology has one switch on the impedance side and four switches on the inverter side. The flowing current through the switches during operating modes is calculated as follows:

48 $$ \left[ \begin{gathered} \,\,\,\,\,\,\,i_{S} \hfill \\ i_{S1} = i_{S4} \hfill \\ i_{S3} = i_{S2} \hfill \\ \end{gathered} \right] = \left[ {\begin{array}{*{20}c} {\mathop 1\limits^{ST} } & {\mathop 0\limits^{non - ST} } & {\mathop 1\limits^{ST} } & {\mathop 0\limits^{non - ST} } \\ 0 & 1 & 0 & 0 \\ 0 & 0 & 0 & 1 \\ \end{array} } \right]\left[ {\begin{array}{*{20}c} {I_{L2} } \\ {I_{o} } \\ {I_{L2} } \\ {I_{o} } \\ \end{array} } \right] $$

Using the above equations, the conduction losses can be expressed as follows:

49 $$ P_{Cond,S} = \frac{1}{{T_{S} }}\int\limits_{0}^{{T_{S} }} {\left( {V_{FS} i_{S} + r_{S} i_{S}^{2} } \right)dt} = V_{FS} D_{ST} \left( {I_{L2} } \right) + r_{S} D_{ST} \left( {I_{L2} } \right)^{2} $$

50 $$ \begin{gathered} P_{Cond,S1} = \cdots = P_{Cond,S4} = \frac{1}{{T_{S} }}\int\limits_{0}^{{T_{S} }} {\left( {V_{FS} i_{S1} + r_{S} i_{S1}^{2} } \right)dt} \\ = 0.5V_{FS} (1 - D_{ST} )\left( {I_{o} } \right) + r_{S} 0.5(1 - D_{ST} )\left( {I_{o} } \right)^{2} \\ \end{gathered} $$

In the above equation, *P*_*Cond,S1*_, *P*_*Cond,S2*_, *P*_*Cond,S3*_, *P*_*Cond,S4*_ and *P*_*Cond,S*_ are the conduction losses of switches *S*_1_, *S*_2_, *S*_3_, *S*_4,_ and *S* respectively.

For simplicity in evaluating the switching losses, the voltage and current waveforms of the switches are assumed to vary linearly during transitions. Under this assumption, the switching losses during the turn-on interval of the switches are calculated as follows:

51 $$ P_{sw,S}^{on} = \frac{1}{{T_{S} }}\int\limits_{0}^{{t_{on} }} {v_{S} } (t) \times i_{S} (t)dt = (\frac{1}{6}BV_{i} I_{L2} )t_{on} f_{S} $$

52 $$ P_{sw,S1}^{on} = \ldots = P_{sw,S4}^{on} = \frac{1}{{T_{S} }}\int\limits_{0}^{{t_{on} }} {v_{S1} } (t) \times i_{S1} (t)dt = (\frac{1}{6}BV_{i} I_{o} )t_{on} f_{S} $$

That *t*_*on*_ is the time when the switch is on.

In addition, the switching losses during the turn-off interval of the switches are calculated as follows:

53 $$ P_{sw,S}^{off} = \frac{1}{{T_{S} }}\int\limits_{0}^{{t_{off} }} {v_{S} } (t) \times i_{S} (t)dt = (\frac{1}{6}BV_{i} I_{L2} )t_{off} f_{S} $$

54 $$ P_{sw,S1}^{off} = \ldots = P_{sw,S4}^{off} = \frac{1}{{T_{S} }}\int\limits_{0}^{{t_{off} }} {v_{S1} } (t) \times i_{S1} (t)dt = (\frac{1}{6}BV_{i} I_{o} )t_{off} f_{S} $$

That *t*_*off*_ is the time when the switch is off.

According to Eqs. (42)–(47), the total switching losses of the proposed topology can be obtained as follows:

55 $$ P_{S,total} = P_{Cond,S1} + \ldots + P_{Cond,S4} + P_{Cond,S} + P_{sw,S1}^{on} + P_{sw,S1}^{off} + \ldots P_{sw,S4}^{on} + p_{sw,S4}^{off} + P_{sw,S}^{on} + P_{sw,S}^{off} $$

###  Diodes losses

Similar to the switches, the diodes experience two types of losses: conduction losses and switching losses. The current flowing through the diodes during the operating modes is determined as follows:

56 $$ \left[ \begin{gathered} i_{D1} \hfill \\ i_{D2} \hfill \\ \end{gathered} \right] = \left[ {\begin{array}{*{20}l} {\mathop 0\limits^{ST} } \hfill & {\mathop 1\limits^{non - ST} } \hfill & {\mathop 0\limits^{ST} } \hfill & {\mathop 1\limits^{non - ST} } \hfill \\ 0 \hfill & 0 \hfill & 0 \hfill & 0 \hfill \\ \end{array} } \right]\left[ {\begin{array}{*{20}c} 0 \\ {I_{L2} } \\ 0 \\ {I_{L2} } \\ \end{array} } \right] + \left[ {\begin{array}{*{20}l} {\mathop 0\limits^{ST} } \hfill & {\mathop 0\limits^{non - ST} } \hfill & {\mathop 0\limits^{ST} } \hfill & {\mathop 0\limits^{non - ST} } \hfill \\ 0 \hfill & 1 \hfill & 0 \hfill & 1 \hfill \\ \end{array} } \right]\left[ {\begin{array}{*{20}c} 0 \\ {I_{L1} - I_{o} } \\ 0 \\ {I_{L1} - I_{o} } \\ \end{array} } \right] $$

According to (49), the conduction losses of the diodes can be calculated as follows:

57 $$ \begin{gathered} P_{Cond,D1} = \frac{1}{{T_{S} }}\int\limits_{0}^{{T_{S} }} {\left( {V_{FD} i_{D1} + r_{D} i_{D1}^{2} } \right)dt} \\ = V_{FD} \left[ {(1 - D_{ST} )(I_{L2} )} \right] + r_{D} (1 - D_{ST} )\left( {I_{L2} } \right)^{2} \\ \end{gathered} $$

58 $$ \begin{gathered} P_{Cond,D2} = \frac{1}{{T_{S} }}\int\limits_{0}^{{T_{S} }} {\left( {V_{FD} i_{D2} + r_{D} i_{D2}^{2} } \right)dt} \\ = V_{FD} \left[ {(1 - D_{ST} )(I_{L1} - I_{o} )} \right] + r_{D} (1 - D_{ST} )\left( {I_{L1} - I_{o} } \right)^{2} \\ \end{gathered} $$

where *P*_*Cond,D1*_, *P*_*Cond,D2*_ are the conduction losses of diodes *D*_1_ and *D*_2_ respectively.

When the diodes are in the ON state, the voltage across them is zero; therefore, their conduction losses in this state are also zero. In contrast, when the diodes turn OFF, a reverse-recovery process occurs, during which a reverse-recovery current flows. This process consists of two time intervals, denoted as *t*_*a*_ and *t*_*b*_. During *t*_*a*_, the voltage across the diodes remains zero, while in *t*_*b*_ the diode voltage is given by the following expressions:

59 $$ v_{D1} = - \left( {\frac{{(1 - D_{ST} )V_{i} }}{{D_{ST}^{2} - 3D_{ST} + 1}} + \frac{{V_{i} }}{{D_{ST}^{2} - 3D_{ST} + 1}}} \right) = (2 - D_{ST} )BV_{i} $$

60 $$ v_{D2} = \frac{{V_{i} }}{{D_{ST}^{2} - 3D_{ST} + 1}} = BV_{i} $$

The switching losses in the off-state can also be calculated as follows:

61 $$ P_{sw,D1}^{{}} = \frac{1}{{T_{S} }}\int\limits_{0}^{{t_{b} }} {p_{D1} (t)} dt = \frac{1}{6}(2 - D_{ST} )BV_{i} I_{rr} t_{b} f_{s} $$

62 $$ P_{sw,D2}^{{}} = \frac{1}{{T_{S} }}\int\limits_{0}^{{t_{b} }} {p_{D1} (t)} dt = \frac{1}{6}BV_{i} I_{rr} t_{b} f_{s} $$

That *I*_*rr*_ is a maximum reverse recovery flow. by having (50–55), the total diodes losses can be obtained:

63 $$ P_{D,total} = P_{Cond,D1} + P_{Cond,D2} + P_{sw,D1} + P_{sw,D2} $$

###  Passive components losses

The inductors and capacitors in the proposed topology also contribute to power losses. Based on the capacitor current in each operating mode, the corresponding expressions can be written as follows:

64 $$ \left[ {\begin{array}{*{20}c} {i_{C1} } \\ {i_{C2} } \\ \end{array} } \right] = \left[ {\begin{array}{*{20}l} {\mathop 1\limits^{ST} } \hfill & {\mathop 1\limits^{non - ST} } \hfill & {\mathop 1\limits^{ST} } \hfill & {\mathop 1\limits^{non - ST} } \hfill \\ 0 \hfill & 0 \hfill & 0 \hfill & 0 \hfill \\ \end{array} } \right]\left[ {\begin{array}{*{20}c} {I_{L1} } \\ {I_{L2} - I_{L1} } \\ {I_{L1} } \\ {I_{L2} - I_{L1} } \\ \end{array} } \right] + \left[ {\begin{array}{*{20}l} {\mathop 0\limits^{ST} } \hfill & {\mathop 0\limits^{non - ST} } \hfill & {\mathop 0\limits^{ST} } \hfill & {\mathop 0\limits^{non - ST} } \hfill \\ 1 \hfill & 1 \hfill & 1 \hfill & 1 \hfill \\ \end{array} } \right]\left[ {\begin{array}{*{20}c} {I_{L2} } \\ {I_{L1} - I_{o} } \\ {I_{L2} } \\ {I_{L1} - I_{o} } \\ \end{array} } \right] $$

The losses associated with the inductors and capacitors can be calculated as follows:

65 $$ P_{L1} = r_{L} (I_{L1} )^{2} $$

66 $$ P_{L2} = r_{L} (I_{L2} )^{2} $$

67 $$ P_{C1} = r_{C} \left( {\int\limits_{0}^{{T_{S} }} {i_{C} } dt} \right)^{2} = r_{C} \left( {(I_{L1} D_{ST} ) + (I_{L2} - I_{L1} )(1 - D_{ST} )} \right)^{2} $$

68 $$ P_{C2} = r_{C} \left( {\int\limits_{0}^{{T_{S} }} {i_{C} } dt} \right)^{2} = r_{C} \left( {(I_{L2} D_{ST} ) + (I_{L1} - I_{O} )(1 - D_{ST} )} \right)^{2} $$

**Fig. 24:**
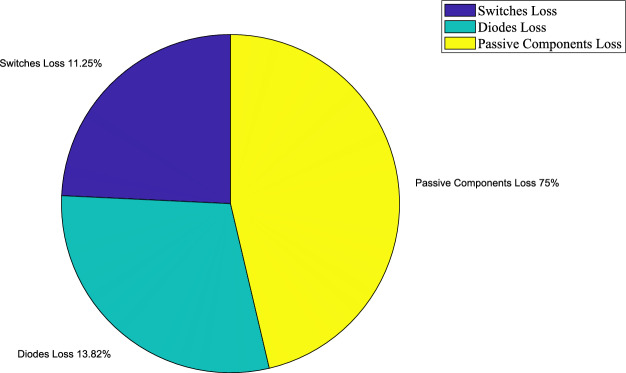
Diagram of losses of different components.

In continuation, by considering the series resistances of the diodes, switches, capacitors, and inductors in efficiency simulation, as well as the forward voltage drops of the switches (approximately 0.5 *V*) and diodes (approximately 0.5 *V*), and the sum of turn-on and turn-off times for the switches (*t*_*on*_ + *t*_*off*_ = *50 ns*), the losses of the different components are first calculated. Then, based on these results, a pie chart illustrating the loss distribution of the various components in the proposed topology is presented in Fig. 24.

As shown in this figure, the passive components account for approximately 75% of the total losses, while the switches contribute about 11.25% and the diodes about 13.82%. Accordingly, the passive-component losses are significantly higher than the losses of the switches and diodes, with the switches exhibiting the lowest share. Considering the operating modes, there are always four passive components—two capacitors and two inductors—present in the current path. Since each of these elements possesses parasitic resistances, their combined contribution results in a considerable portion of the total power losses.

## Conclusion

This paper has presented a new class of active impedance-source inverters that achieve lower voltage stress on semiconductor devices and reduced component count compared to conventional active Z-source inverters. These improvements lead to smaller size, lower volume, and potentially reduced cost while maintaining high performance. The proposed topologies were analyzed in detail, covering their operating modes, boost factor, voltage and current stresses, and design considerations for passive and active components. A comprehensive comparative study with several conventional topologies demonstrated that the proposed designs exhibit lower total blocked voltages across active switches, diodes, and capacitors, reduced volumetric size of passive components, comparable or superior efficiency under various load conditions, and low sensitivity of the boost factor to duty cycle variations, which simplifies practical control. Experimental results from a hardware prototype confirmed the accuracy of the analytical equations and validated the performance improvements.

Beyond the technical evaluation, the proposed inverters are well suited for non-grid-connected industrial applications. They are particularly advantageous in systems that demand high-frequency, adjustable output voltages without strict harmonic constraints. Typical examples include electrochemical processes such as electroplating or electrolysis, where high-frequency pulsed voltages enhance reaction efficiency and improve deposition quality. Induction heating systems are another relevant case, since they depend on high-frequency waveforms to generate localized heating through induced currents. Importantly, these applications do not require a common ground between the input and output, which further supports the suitability of the proposed inverters. The controllable boost factor and high-frequency operation make these topologies highly adaptable to processes that demand precise voltage regulation, compact design, and high efficiency without the use of additional bulky filtering stages.

## Data Availability

All data generated and analyzed during the current study are available in the manuscript.
